# Smart Nanoparticle‐Based Platforms for Regulating Tumor Microenvironment and Cancer Immunotherapy

**DOI:** 10.1002/adhm.202202063

**Published:** 2022-12-20

**Authors:** Ruoyu Cheng, Hélder A. Santos

**Affiliations:** ^1^ Department of Biomedical Engineering University Medical Center Groningen University of Groningen Ant. Deusinglaan 1 Groningen 9713 AV The Netherlands; ^2^ W. J. Kolff Institute for Biomedical Engineering and Materials Science University Medical Center Groningen University of Groningen Ant. Deusinglaan 1 Groningen 9713 AV The Netherlands; ^3^ Drug Research Program Division of Pharmaceutical Chemistry and Technology Faculty of Pharmacy University of Helsinki Helsinki FI‐00014 Finland

**Keywords:** cancer immunotherapy, clinical translation, drug delivery, nanoparticles, tumor microenvironment

## Abstract

Tumor development and metastasis are closely related to the tumor microenvironment (TME). Recently, several studies indicate that modulating TME can enhance cancer immunotherapy. Among various approaches to modulating TME, nanoparticles (NPs) with unique inherent advantages and smart modified characteristics are promising candidates in delivering drugs to cancer cells, amplifying the therapeutic effects, and leading to a cascade of immune responses. In this review, several smart NP‐based platforms are briefly introduced, such as responsive NPs, targeting NPs, and the composition of TME, including dendritic cells, macrophages, fibroblasts, endothelial cells, myeloid‐derived suppressor cells, and regulatory T cells. Moreover, the recent applications of smart NP‐based platforms in regulating TME and cancer immunotherapy are briefly introduced. Last, the advantages and disadvantages of these smart NP‐based platforms in potential clinical translation are discussed.

## Graphical abstract

Smart nanoparticle‐based platforms can arrive at the tumor site through active/passive targeting delivery, and respond to external stimulations, such as acid tumor microenvironment, leading to the release of payloads or other physiochemical changes. Such nanoparticles can regulate the tumor microenvironment and cancer immunotherapy via interacting with infiltrating immune cells, fibroblasts, endothelial cells, myeloid‐derived suppressor cells, extracellular matrix, and secreted cytokines.

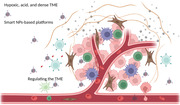

## 1 Introduction

Unlike normal tissues, tumors are famous for specific characteristics, such as acid, hypoxic, and dense microenvironment. These characteristics can be caused by uncontrolled proliferation, insufficient oxygen supply, limited waste removal, and poor nutrition penetration. Besides the specific characteristics, the tumor microenvironment (TME) consists of not only cancer cells but also other cells, such as T cells, macrophages, dendritic cells, fibroblasts, endothelial cells, secreted cytokines, and extracellular matrix.^[^ ^1^ ^]^ Together, these elements influence the prognosis and therapeutic efficacy of the tumor. For example, the acid and hypoxic TME can promote the mutations of cancer cells, leading to metastasis; the dense TME would limit the infiltration of immune cells and therapeutic drugs. Recently, many studies have indicated that TME can be either hot or cold according to the infiltration of immune cells. The hot TME or tumor means the tumor infiltrated with a large amount of inflammatory immune cells, such as M1 type macrophages and cluster of differentiation (CD)8^+^ T cells; the cold TME or tumor means the tumor infiltrated with plenty of suppressive immune cells, such as M2 type macrophages, regulatory T cells (Treg), and myeloid derived suppressor cells (MDSCs). In addition, hot TME is always associated with a promising prognosis, prolonged survival period, and increased therapeutic effects of immunotherapy compared to cold TME.^[^ ^2^ ^]^ Therefore, regulating the TME, especially converting the cold TME to the hot TME, becomes a challenge for cancer treatment, particularly cancer immunotherapy.

Compared to traditional medicines such as capsules and tablets, nanomedicines are famous for their multi‐functionalities and intelligent properties, which means that nanomedicines can make treatments more precise and effective. With the development of nanomedicines, increasing smart nanoparticles (NPs) are well designed with various characteristics, such as the large specific surface area, tailorable physicochemical properties, stimulation‐responsive release of payload, stimulation‐induced aggregation, active targeting capability, and photothermal and photodynamic properties.^[^ ^3^ ^]^ Considering the specific characteristics, such as acid pH, hypoxic environment, increased glutathione (GSH) concentration, and accumulated lactose in TME, increasing NPs‐based strategies are proposed to regulate the TME. This is because these specific characteristics can be used to trigger the precise drug release or other therapeutic treatments for improved treatment efficiency.^[^ ^4^ ^]^

Herein, in this review, we will first introduce various types of responsive or targeting NPs. Then, we will introduce the compositions of TME and how these compositions would influence the TME. Afterward, we will give examples of how nanoparticles can be applied in regulating the TME and cancer immunotherapy. Last, we analyze the challenges and future perspectives in the field. The smart NPs‐based platforms discussed in this review are at the interfaces of nanomedicines, TME, and pharmaceutical science and will appeal to a broad readership and inspire future developments for cancer immunotherapy.

## 2 Smart Nanoparticle‐Based Platforms

Particles with diameter lower than 100 nm are usually denoted as nanoparticles (NPs). NPs can be commonly prepared by bottom–up methods, which means they can be fabricated from atoms and molecules. In addition, NPs can be constructed by top–down methods, indicating that the bulk materials can be processed into NPs. Unlike atoms, molecules, and bulk materials, NPs exhibit new properties, such as increased surface energy and abundant reactive centers, which are attributed to the volume effect and surface (interface) effect.^[^ ^5^, ^6^ ^]^ These unique properties make NPs easily tailored with controlled physicochemical properties, such as surface areas, hydrodynamic diameters, and surface zeta‐potential. In recent decades, NPs have attracted wide attention from various fields, especially nanomedicines.^[^ ^7^ ^]^

**Scheme 1:**
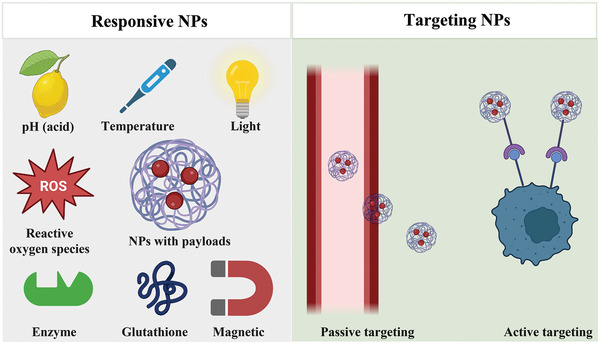
The scheme of various stimulations‐responsive NPs, such as pH, temperature, light, reactive oxygen species, enzyme, glutathione, and magnetic responsive NPs, and the targeting NPs, such as passive targeting NPs due to the enhanced permeability and retention (EPR) effects and active targeting NPs due to the ligands–receptor interaction. Created with BioRender.com.

In nanomedicine, NPs exhibit unique advantages. For example, the NPs (diameter lower than 200 nm) tend to accumulate in tumor areas due to the enhanced permeability and retention effect; NPs modified with specific ligands can target specific cells or even organelles; NPs consisting of responsive materials can respond to various simulations, such as pH, temperature, light, magnetism, and enzyme.^[^ ^8^, ^9^, ^10^ ^]^ With these advantages, NPs are widely used as therapeutic agent carriers, diagnostic probes, contrast reagents, and vaccines, as nanomedicines.^[^ ^11^, ^12^ ^]^ This section will systematically introduce various responsive NPs, and active and passive targeting NPs, as shown in **Scheme** **1**.

### 2.1 Responsive NPs

Some stimulation‐responsive materials are widely applied in synthesizing NPs to make these NPs smarter with enhanced therapeutic effects (**Table** **1**). Under external stimulations, NPs properties, structure, interactions, and dimension will change, leading to rearrangement or changes in their aggregation state. As shown in Table 1, some common materials are applied for different responsive purposes. In the following sections, we will, respectively introduce these responsive NPs.

**Table 1:** Brief summary of responsive NPs

Stimulation	Materials	Ref.
pH	*β*‐amino esters, imidazole groups	[13, 14]
Temperature	Pluronic tri‐block copolymer, poly (n‐isopropyl acrylamide)	[16, 17]
Light	Octa‐triethoxysilylated Zn phthalocyanine, *π*‐conjugated oligomer, merocyanine 540	[18, 19, 20]
Magnetic	Fe_3_O_4_, lanthanide	[22, 23]
Enzyme	PLGVRK peptides, *γ*‐glutamyl moieties	[25, 26]
Reactive oxygen species	Ferrocene, thioketal	[28, 29]
Glutathione	Pt(IV) prodrugs, homodithiacalix[4]arene	[30, 31]
MMP	MT1‐AF7p peptide	[33]

#### 2.1.1 pH‐Responsive NPs

Herein, pH‐responsive NPs mean the acid‐responsive NPs. Under the acid environment, NPs can change hydrodynamic diameters, surface zeta‐potential, morphologies, or release cargo. The phenomenon could be attributed to the acid‐cleaved bonds or charge shifting.

Acid cleaved bonds are stable at neutral pH but cleaved at acidic pH. For example, poly (*β*‐amino ester) obtained by regular Michael‐addition reaction can be effectively cleaved, leading to the dissociation of the polymeric structures and release of payloads. Wang et al. prepared a new style of acid‐responsive covalent organic polymers (COPs) by using acryloyl meso‐tetra(p‐hydroxyphenyl) porphine (acryloyl‐THPP) to react with 4,4′‐trimethylene dipiperidine to form the acid‐responsive cross‐linked biodegradable *β*‐amino esters (BAEs). The BAEs‐modified COPs were investigated in acid‐induced doxorubicin (DOX) release (≈50% released DOX in COPs with BAEs compared to lower than 20% released DOX in COPs without BAEs at 24 h).^[^ ^13^ ^]^

**Figure 1:**
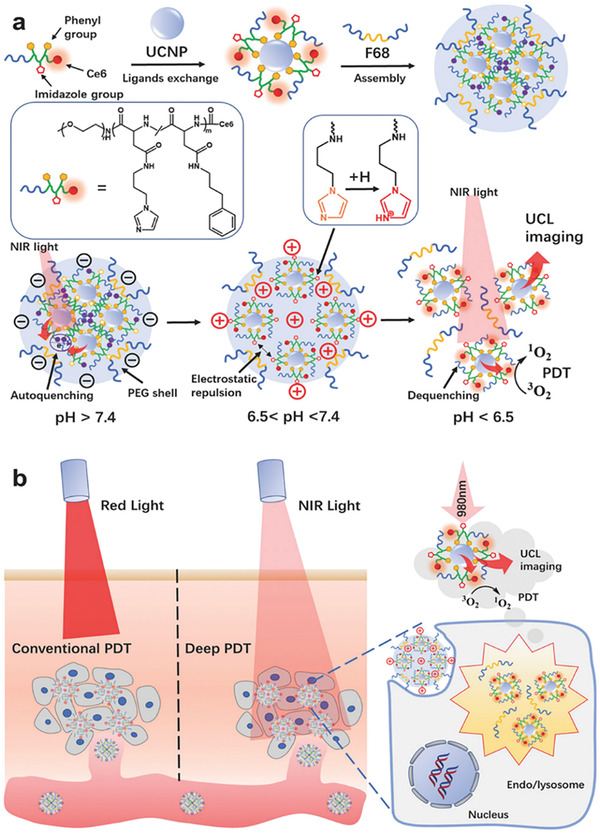
Schematic of a) preparing the pH‐responsive ligand‐assisted assembly of UCNPs and b) the tumor‐pH‐responsive deep tissue photodynamic therapy. Reproduced with permission.^[^ ^14^ ^]^ Copyright 2018, Wiley‐VCH GmbH.

Some functional groups are a negative charge at neutral pH but a reverse charge at acid pH. As shown in **Figure** **1**, Li et al. developed acid‐responsive photodynamic nanoagents (UCNPs) by utilizing the ionization of the imidazole groups (pKa ≈ 6.8); the nanoagents change negative zeta potential (≈ −15 mV at pH 7.4 to +10 mV at pH 6.5, and to +30 mV at pH 5.5). The reverse charge not only increases cell uptake efficacy but also raises the electrostatic repulsive force among NPs.^[^ ^14^ ^]^

#### 2.1.2 Temperature Responsive NPs

The phase behavior of temperature‐responsive NPs is critical in determining the physicochemical and biological properties under certain conditions.^[^ ^15^ ^]^ For example, Vaghasiya et al. prepared a pluronic tri‐block copolymer (PTBC) with a reversible temperature‐sensitive phase transformation in water. The PTBC was easily dispersed in a water medium below their critical micelle temperature (CMT) but precipitated above the CMT. When the temperature was raised (≈25 °C) above the CMT, the PTBC dehydration and stronger interactions between the polymer blocked themselves, such as poly (propylene oxide) and poly (ethylene oxide), trapping the targeted objectives into the PTBC matrix.^[^ ^16^ ^]^

As another critical property for some temperature‐responsive materials, lower critical solution temperature (LCST) is the temperature below which the materials completely solvate in the aqueous phase; above this temperature, materials become insoluble and phase‐separated. Taking advantage of LCST, Chen et al. first prepared temperature‐responsive photoacoustic contrast agents consisting of poly (n‐isopropyl acrylamide) (PNIPAM) that could rapidly reduce their volume when the surrounding temperature was higher than their LCST. In contrast, when the surrounding temperature was lower than their LCST, the agents could expand and resume their initial volumes. These properties made the agents work in both first (650–950 nm) and second (1000–1700 nm) near‐infrared optical windows of tissue with suppressing the background signals and an additional increase of more than fivefold in imaging contrast in vivo.^[^ ^17^ ^]^

#### 2.1.3 Light‐Responsive NPs

Herein, the light‐responsive properties indicate the photoactivation with the energy conversion processes, such as photochemical, photothermal, and photosensitization. Ekineker et al. prepared mesoporous organosilica NPs (PHT‐PMO) consisting of octa‐triethoxysilylated Zn phthalocyanine precursor. The PHT‐PMO NPs exhibited limited cytotoxicity under the dark surrounding but high phototoxicity when irradiated at 650 nm. In addition, remarkable near‐infrared phototoxicity was observed when the NPs were excited at 760 and 810 nm. Furthermore, the NPs were aminated to promote electrostatic complexation with siRNA, and efficient photochemical internalization was observed on MCF‐7 cancer cells.^[^ ^18^ ^]^

Li et al. developed a self‐assembly system with F8‐IC and polyethylene glycol (PEG) *π*‐conjugated. The system exhibited a high photothermal conversion efficiency of 82%, heating water to more than 65 °C in 300 s. In addition, the tumor tissues were heated to more than 60 °C in 300 s in the 4T1‐tumor‐bearing nude mice model.^[^ ^19^ ^]^ Ding et al. synthesized large‐pore mesoporous‐silica‐coated upconversion NPs (UCMSs) by a typical silica sol–gel reaction using mesitylene as a pore‐swelling agent. The high loading efficacy of photosensitizers merocyanine 540 (MC540), chicken ovalbumin (OVA), and tumor cell fragment were observed in the UCMSs. Moreover, under the 980 nm near‐infrared irradiation, the strongest type 1 T helper (Th1) and type 2 T helper (Th2) immune responses and the highest frequency of CD4^+^, CD8^+^, and effector memory T cells were observed in the colon cancer (CT26)‐tumor‐bearing BALB/c mice.^[^ ^20^ ^]^

#### 2.1.4 Magnetic Responsive NPs

Under the guidance of an applied magnetic field, the magnetic responsive NPs can be spatially and temporally controlled; NPs can be externally operated in the system, providing a non‐invasive approach to remote control.^[^ ^21^ ^]^ Demin et al. prepared NPs with core–shell structure; the Fe_3_O_4_ magnetic NPs were used as a core structure, then, the silica covalently modified by [(3‐triethoxysilyl)propyl]succinic acid–polyethylene glycol (PEG 3000) conjugates were the shell structure. The NPs with high loading efficiency of DOX could increase the release of DOX under the action of an alternating magnetic field in acidic medium compared to the neutral medium without alternating magnetic field.^[^ ^22^ ^]^ Wang et al. coated magnetic lanthanide‐doped upconversion NPs (UCNPs) with liquid marbles, which can convert near‐infrared light into visible light. In addition, the NPs can repeat tip opening and move under the magnetic direction, providing a promising miniature reactor for photodynamic therapy of cancer cells.^[^ ^23^ ^]^

#### 2.1.5 Enzyme Responsive NPs

Enzymes are various types of bioactive products that can accelerate chemical reactions. Typically, there are several enzyme stimulations, such as lipase, matrix metalloproteinase (MMP), cathepsin, glycosidase, and oxidoreductase enzyme, that can be applied in designing the enzymes responsive NPs.^[^ ^24^ ^]^ For example, Brave et al. prepared cabazitaxel‐loaded micelle consisting of cholesterol‐PLGVRK (as an enzyme‐responsive peptide)‐PEG_2000_ and cholesterol‐(2‐[3‐(1,3‐dicarboxypropyl) ureido] pentanedioic acid (DUPA, as a targeting ligand)‐PEG_3000_, with low critical micelle concentration (5.7 µg mL^−1^), high drug loading (43.77%), and high entrapment efficiency (79.69%). These micelles were cleaved by the MMP2 that was abundant in the prostate TME, leading to the burst cabazitaxel release.^[^ ^25^ ^]^ Due to the overexpressed membrane *γ*‐glutamyl transpeptidase (GGT) on the external surface of endothelial cells and metabolically active tumor cells in the peripheral blood vessels, Zhou et al. prepared a GGT‐cleaved camptothecin–polymer conjugate that actively infiltrated throughout the tumor tissue through transcytosis. The conjugate exhibited significant inhibition of pancreatic tumor growth by eradicating small solid tumors (−100 mm^3^) and regressing large established tumors (−500 mm^3^). In addition, the conjugate significantly extended the survival of orthotopic pancreatic tumor‐bearing mice compared to that of the first‐line chemotherapeutic drug gemcitabine.^[^ ^26^ ^]^

#### 2.1.6 Reactive Oxygen Species Responsive NPs

Reactive oxygen species (ROS), such as superoxide anion (O_2_^−^), hydroxyl radical (˙OH), and hydrogen peroxide (H_2_O_2_), are commonly generated as side products during the aerobic metabolic process. However, exposure to ionizing radiation or pathogens increases intracellular ROS levels. In addition, some pathological changes, such as inflammation, infection, and tumor, are accompanied by increased intracellular ROS levels.^[^ ^27^ ^]^ Therefore, this characteristic is commonly used in designing ROS‐responsive NPs for treating tumors. Qin et al. prepared the NPs platform via the host–guest interaction between chlorin e6‐conjugated *β*‐cyclodextrin (Ce6‐CD) and ferrocene‐modified FFVLG3C‐PEG conjugates (Fc‐pep‐PEG). Under theTME, the hydrophobic Fc was oxidized to hydrophilic Fc^+^ by the ROS, leading to the disassembling NPs. Due to the intermolecular hydrogen effects, these Fc^+^‐pep‐PEG fragments formed nanofibers by self‐assembly with prolonged local retention in the tumor site.^[^ ^28^ ^]^ Although the tumor cell had a higher ROS level (up to 10 × 10^−5^ m) than the normal cell (≈20 × 10^−9^ m), it would be helpful if the intracellular ROS level could be further improved in cancer cells, leading to the tumor cell apoptosis. Lv et al. covalently conjugated *α*‐Tocopheryl succinate (*α*‐TOS) with mPEG2k by the ROS‐cleavable thioketal (TK) linker. The *α*‐TOS could be released in the tumor site due to the cleaving of the TK linker, which inhibited the complex II in the mitochondrial respiratory chain and amplified the ROS level in tumor cells, causing the tumor cell apoptosis.^[^ ^29^ ^]^

#### 2.1.7 Glutathione Responsive NPs

Glutathione (GSH) is mainly responsible for regulating the cellular redox state, protecting the cell from damage caused by ROS and xenobiotics. In addition, the GSH can be protective and pathogenic because the tumor has a higher GSH level than the normal tissues contributing to tumor metastasis and chemotherapy resistance. Considering the high GSH level in tumor cells, Ling et al. fabricated a GSH‐responsive NPs system consisting of Pt(IV) prodrugs and PEG. The NPs can be internalized into tumor cells through the macropinocytosis effects and disintegrated by the intracellular GSH, leading to the release of Pt(II) ions, which ultimately cause the mitochondria‐mediated apoptosis of cisplatin‐resistant tumors.^[^ ^30^ ^]^ Due to the presence of disulfide‐bridges, the homodithiacalix[4]arene (HDT‐C4A) can be degraded by the GSH. Cheng et al. prepared the paclitaxel (PTX)‐loaded HDT‐C4A NPs by the oil‐in‐water (o/w) emulsion method with high drug loading efficiency (51.3%). The NPs could specifically release PTX in the tumor cells with high GSH levels, inhibiting more than 50% cell viability of MCF‐7 cells. Meanwhile, the NPs had limited influence on the cell activity of normal cells, inhibiting less than 30% cell viability of RAW264.7 cells.^[^ ^31^ ^]^

#### 2.1.8 MMPs Responsive NPs

MMPs are proteolytic enzyme families degrading multiple components of the extracellular matrix. In addition, MMPs also participate in tumor invasion, neoangiogenesis, and metastasis, which is regarded as a typical pharmacologic target for cancer treatment.^[^ ^32^ ^]^ Gu et al. synthesized an MT1‐AF7p peptide presenting high binding affinity to membrane type‐1 MMP (MT1‐MMP) that is overexpressed on both angiogenic blood vessels and glioma cells. Then, they modified PTX‐loaded PEG‐polylactic acid (PLA) NPs with the peptide; a tumor‐homing and penetrating peptide iRGD was modified on the NPs. The NPs effectively penetrated the glioma spheroids and significantly inhibited the growth of glioma spheroids. Moreover, the most prolonged survival period was also observed in the C6 glioma‐bearing mice treated with NPs.^[^ ^33^ ^]^

#### 2.1.9 Multiple Responsive NPs

Besides responding to a single stimulation, some innovative NP systems can simultaneously or progressively respond to multi stimulations, such as two stimulations (pH and light) and three stimulations (pH, light, and enzymes), which make these NP systems more powerful than the NPs responding to single stimulation. Taking advantage of enzyme‐induced size‐reduction and the reactive‐oxygen‐species‐driven deselenization, Wei et al. fabricated a DOX‐loaded self‐assembling selenopeptide consisting of Arg‐Gly‐Asp (RGD), MMP‐2 cleavable linker (PLGVR), and a reactive oxygen species (ROS)‐responsive N‐terminal Se‐dodecyl‐selenocysteine‐containing double‐chained tail (Sec (Dod)2K). The NPs were cleaved into small fragments under the stimulation of MMP2 enzymes; in addition, the loaded DOX could be dramatically released (80% DOX was released within 4 h) under the simulation of MMP2 enzymes and H_2_O_2_. DOX‐induced chemotherapy and selenopeptide‐induced immunotherapy were tested in the orthotopic MDA‐MB‐231 tumor model in mice.^[^ ^34^ ^]^ Li et al. synthesized photothermal agents that could be transformed from aggregated state to a dispersed state by boosting photothermal conversion efficiency (PCE). Under the pH stimulation, this transformation was achieved by releasing photothermal molecules from NPs, with increased PCE from 43% to 60%, by changing the pH values from 7.4 to 5.0. In addition, both in vitro and in vivo experiments demonstrated that this transformation appeared in the lysosome with the acid stimulation, providing a potential candidate for the photothermal therapy technique.^[^ ^35^ ^]^

Triple responsive NPs also attract wide attention from researchers. Tang et al. fabricated pH/glutathione (GSH)/ ROS triple‐responsive PEG‐PPMDT NPs. The NPs can rapidly release artemether under the synergistic stimulation of acidic endoplasmic pH and high intracellular GSH/ROS in HepG2 cancer cells. In addition, the produced ROS can be further magnified by the released artemether using the interactions between the formed Fe^2+^ ions at acid pH and external ultrasound irradiation. Taken together, the NPs can enhance the chemo‐sonodynamic therapy with 40% eliminated HepG2 tumors in nude mice.^[^ ^36^ ^]^

### 2.2 Targeting NPs

Drug treatment is still a practical approach for most diseases, especially cancer. After intravenous injection, only a limited amount of drug arrives at the tumor tissues. Most of the drug is metabolized by the other organs, leading to unsatisfactory therapeutic and side effects. Moreover, the drug concentration in tumor tissues is predominantly determined by the local blood flow at the tumor site. Under the relatively stable blood flow, the patient must be administered with a high dose of the drug and with frequent injections to reach the satisfactory therapeutical window at the tumor site, which lead to the unspecific exposure of drugs in healthy organs, causing severe side effects. Therefore, researchers have developed various strategies to increase the concentration of therapeutic agents at the tumor site.

#### 2.2.1 Active Targeting NPs

By taking advantage of the affiliation between ligands and receptors, active targeting NPs aim to increase the concentration of NPs and therapeutic agents in the targeted tissues, cells, or organelles. There are some most used targets in designing active targeting NPs, such as vascular endothelial growth factor receptors (VEGFRs), *α*v*β*3 integrin, vascular cell adhesion molecule‐1 (VCAM‐1, CD106), human epidermal receptor (HER), transferrin receptors (CD71), and folate receptors (**Table** **2**).

**Table 2:** Brief summary of common targets in designing active targeting NPs

Target	Locations	Ref.
VEGFRs	Endothelial cells	[37]
*α*v*β*3 integrin	Cancer cells, such as B16F10, U87MG, MDA‐MB‐231, 4T1, A2780, OVCAR‐3, and A549 cells	[38, 39, 40, 41, 42]
VCAM‐1 (CD106)	Endothelial cells	[43, 44]
HER	Cancer cells, such as breast and gastroesophageal cancer cells	[45, 46]
Transferrin receptor (CD71)	Cancer cells, red blood cells, and brain endothelial cells	[47, 48]
Folate receptors	Epithelial cancers, such as breast, cervical, colorectal, renal, nasopharyngeal, ovarian, and endometrial cancer cells	[49, 50]

Angiogenesis is a typical pathological process in many solid cancers and vascular and endothelial growth factor contributes to this process. Chen et al. prepared anti‐VEGFR‐2 modified phthalocyanine zinc (ZnPc) and perfluorohexane loaded poly(lactic‐co‐glycolic acid) (PLGA) NPs with enhanced ultrasound (US) and photoacoustic (PA) image. Due to the active targeting capability of anti‐VEGFR‐2, increased concentration of NPs can significantly convert light energy to heat in the tumor, which could be a potential candidate for dual‐modality imaging and photothermal therapy.^[^ ^37^ ^]^

The *α*v*β*3 integrin is overexpressed in various cancer cells, such as B16F10, U87MG, MDA‐MB‐231, 4T1, A2780, OVCAR‐3, and A549 cells.^[^ ^38^, ^39^, ^40^, ^41^ ^]^ As one typical ligand for the *α*v*β*3 integrin, RGD is widely applied in fabricating NPs. Wu et al. entrapped the enzyme catalyst glucose oxidase (GOD) and H_2_O_2_‐sensitive molecule manganese carbonyl (MnCO) to biodegradable hollow mesoporous organosilica NPs (HMONs) modified with RGD for targeting the *α*v*β*3 integrin‐overexpressed MDA‐MB‐231 cells. Under the therapeutic effect, the survival rate of MDA‐MB‐231 bearing nude mice was improved to 100% on day 60.^[^ ^42^ ^]^

VCAM‐1 is also known as cluster of differentiation 106 (CD106), a cell adhesion molecule. In addition, the expression of VCAM‐1 is related to oncogenesis, tumor angiogenesis, and metastasis in gastric carcinoma.^[^ ^43^ ^]^ Since most tumors are related to inflammation, inflamed endothelial cells up‐regulate the expression of VCAM‐1. Park et al. genetically modified plasma membranes from C1498 cells with very late antigen–4 (VLA‐4); then, the PLGA cores were coated with these membranes to prepare the NPs. With the VLA‐4, the binding efficacy between NPs and C1498‐VCAM cells improved three times that with NPs and C1498‐WT cells. In addition, after loading with dexamethasone, the NPs also exhibited significant therapeutic efficacy on the inflamed tissues.^[^ ^44^ ^]^

HER is a member of the epidermal growth factor receptor (EGFRs) family with tyrosine kinase activity. The dimerization of the HER initiates a series of signaling pathways, causing cell proliferation and tumorigenesis. In addition, 15–30% of breast cancer and 10–30% of gastroesophageal cancers are related to the overexpression of HER2.^[^ ^45^ ^]^ Liu et al. constructed raspberries‐like NPs with ultrasmall gold nanospheres (UGNPs) and an MMP‐2/9‐sensitive peptide as a cross‐linking agent; the NPs were further modified with trastuzumab (TRA) and mertansine derivatives (DM1) via the Au—S bond. TRA was used as a HER targeting ligand in the NPs, and the Au—S bond could be replaced by the sulfhydryl group of GSH, leading to the GSH‐responsive drug release. The NPs could effectively penetrate and inhibit the SK‐BR‐3 tumor spheroid. With the help of TRA, the concentration of TRA‐NPs was 11.09 times higher than NPs without TRA.^[^ ^46^ ^]^

**Figure 2:**
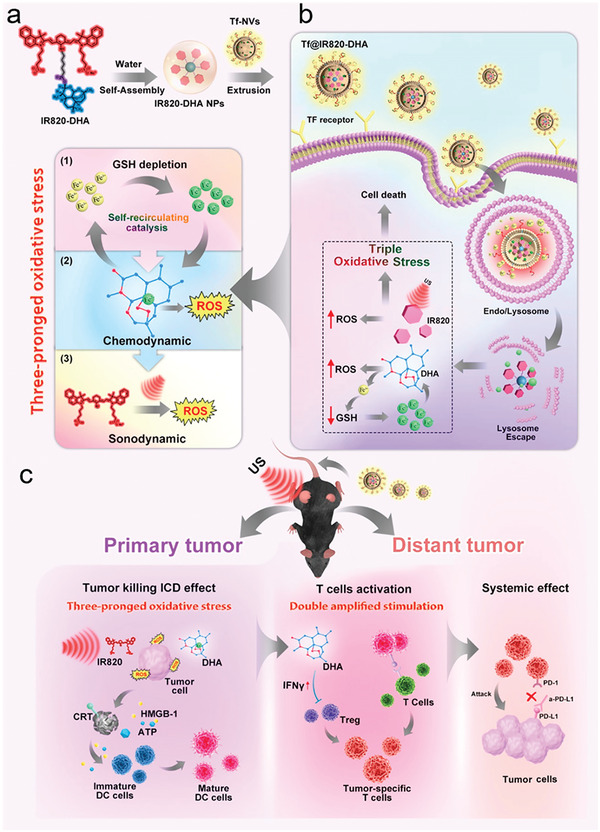
Schematic depiction of a) Preparing the Tf@IR820‐DHA, b) the mechanism of Tf@IR820‐DHA NV mediates three‐pronged oxidative stress on tumor cells, and c) the Tf@IR820‐DHA NV mediate tumour killing immunogenic cell death effect, causing the cascading T cells activation and systemic effects. Reproduced with permission.^[^ ^48^ ^]^ Copyright 2022, American Chemical Society.

Transferrin receptor (CD71) is a membrane glycoprotein that mediates the cellular uptake of iron from plasma glycoprotein. In addition, transferrin receptors are more widely expressed in cells with activated proliferation than in quiescent cells.^[^ ^47^ ^]^ Therefore, some researchers regard the transferrin receptor as a tumor‐targeting receptor. As shown in **Figure** **2**, Bai et al. genetically engineered transferrin expressed on 293T cells; then, these cell membranes were coated on the IR820‐dihydroartemisinin to prepare the NPs (Tf@IR820‐DHA). The NPs exhibited targeting ability due to the transferrin, followed by the programmable catalysis, sonodynamic effect, and excellent fluorescent/photoacoustic imaging capacity, which boosted a programmed death‐ligand 1(PD‐L1)‐mediated immune checkpoint blocking under synergetic triple stimuli‐activated oxidative stress‐associated immunogenic cell death in the mice Hep1‐6 tumor model.^[^ ^48^ ^]^

Folic acid is essential in cellular metabolism, DNA synthesis, and repair. The transportation of folic acid mainly depends on the folate receptors (FR), especially FR‐*α*, which is widely expressed in tumor cells with limited expression in normal cells.^[^ ^49^ ^]^ Zeng et al. first prepared the MIL‐88B(Fe) consisting of metal ions (Fe^3+^) and 2‐aminoterephthalic acid (NH2‐BDC); then, the MIL‐88B was reacted with 2‐methylimidazole and zinc ions (Zn^2+^) to form ZIF8 on the surface of MIL‐88B, followed by loading with DOX, MnOx, and modifying with folic acid. With the help of folic acid, accelerated endocytosis of NPs was observed on both HepG‐2 cells and hCMEC/D3 cells.^[^ ^50^ ^]^

#### 2.2.2 Passive Targeting NPs

The passive targeting strategy is the most applied approach in clinical nanomedicine by taking advantage of the abnormal physiological environments in the tumor, such as abnormal vascularization, loose structure of tumor vascular endothelium, and so on.^[^ ^51^ ^]^ So far, various commercial passive targeting nanoparticles have been applied in clinical, such as Genexol, Abraxane, Marqibo, Onivyde, and NBTXR3.^[^ ^52^ ^]^

One of the mechanisms behind the passive targeting strategy is the enhanced permeability and retention (EPR) effect. NPs with a hydrodynamic diameter lower than 200 nm can passively accumulate in the tumor due to the EPR effect. According to this mechanism, researchers further explored the balance and efficacy between long circulation and effective accumulation. The modification of PEG can prolong the circulation time of NPs and PEG with different molecules can increase the hydrodynamic diameter of NPs to a different extent. To explore the size‐dependent EPR effect, H. Kang et al. prepared PEG NPs with different PEG molecules at 1, 5, 10, 20, 40, and 60 kDa). Smaller‐sized PEG NPs (≤20 kDa, 12 nm) exhibited significant passive tumor targeting on the Hela‐bearing mice model. In contrast, the larger‐sized PEG NPs (40 and 60 kDa) exhibited prominent accumulation on the main organs, such as the lungs, liver, and pancreas. They concluded that PEG NPs with 20 kDa molecules exhibited the highest tumor accumulation, which could be a promising platform for the engineered NPs in tumor treatments.^[^ ^53^ ^]^

Moreover, some researchers developed various strategies attempting to amplify the EPR effect. Awaad et al. fabricated a polyzwitterion based on ethylenediamine‐based carboxybetaine [PGlu (DET‐Car)], which could change the net charge, according to the surrounding pH. Under the physiological pH, the net charge was neutral, allowing the prolonged NPs’ blood circulation. In contrast, the net charge changed to be cationic with enhanced tissue adhesion under the acid tumor microenvironment, which was attributed to the stepwise protonation behavior of ethylenediamine. Interestingly, the PGlu (DET‐Car)‐coated NPs penetrated the deeper tumor with high accumulation in the hypoxic area, which could be attributed to the hypoxic area being more acidic than the non‐hypoxic area, causing dramatically cationic net charge of PGlu (DET‐Car)‐coated NPs.^[^ ^54^ ^]^

Rou et al. designed a responsive aggregated nanoplatform loaded with DOX and hydroxychloroquine (HCQ) (AuNPs‐D&H‐R&C) for combing the chemotherapy, inhibiting autophagy, and reprogramming macrophages in one platform. Through the EPR effect, the NPs passively targeted the tumor site. After the trigger of overexpressed furin in MCF‐7/ADR cells, the NPs further aggregated the tumor site. After exposure to acid endosomes/lysosomes, HCQ was released, and it inhibited the autophagy in MCF‐7/ADR cells, restoring tumor cells' sensitivity to DOX. In addition, the inhibited autophagy also reprogrammed the M2 macrophages to M1 macrophages, presenting a synergistic effect in restoring the tumor resistance of DOX.^[^ ^55^ ^]^

### 2.3 Summary

By taking advantage of tumor physiological characteristics, such as acid pH, elevated GSH concentration, high autophagy level, hypoxia, dense extracellular matrix (ECM), abnormal vascularization, loose structure of tumor vascular endothelium, and specific tumor surficial antigens, various NP platforms, such as the responsive NPs, and active and passive targeting NPs, were designed for improving the therapeutic effects or image quality in cancer treatments.

However, some challenges are hindering the application of NPs in clinical settings. For example, although the targeting strategies can improve the NPs concentration in the tumor site, there is still limited drug in the tumor compared to the encapsulated drug amount; When the NPs circulate in the blood, abundant proteins, such as serum albumin and fibrinogen, form the protein corona on the surface of NPs. Then, the formed protein corona can block the interaction between NPs and cell membrane, leading to decreased internalization and targeting capability. As for the active targeting NPs, the potential off‐target effects can cause unsatisfactory therapeutic or undesired side effects. The unsatisfactory reproducibility of NPs, especially some tailored NPs, would be a challenge, but hopefully, a more reliable and smart NPs system can be developed in the future.

## 3 Tumor Microenvironment

**Scheme 2:**
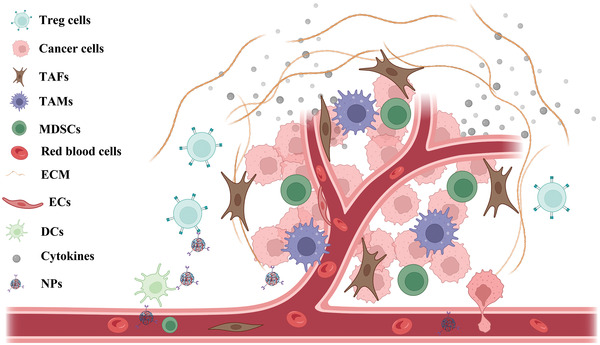
The scheme of complex TME consisting of cancer cells, Treg cells, TAFs, TAMs, MDSCs, ECs, DCs, dense ECM, and cytokines. Created with BioRender.com.

The tumor consists of not only cancer cells but also infiltrating immune cells, fibroblasts, endothelial cells, myeloid‐derived suppressor cells, extracellular matrix, and secreted cytokines, as shown in **Scheme** **2**.^[^ ^56^ ^]^ Besides the cancer cells, other components in a tumor consist of theTME. The cancer cells stimulate the TME changes in the host tissues to support tumor proliferation, differentiation, and metastasis. In the beginning, the interaction between cancer cells and TME benefits cancer cell survival, drug resistance, and proliferation. Furthermore, hypoxic and acid TME are gradually generated with the rapid growth of tumors. Therefore, the tumor develops enhanced abnormal angiogenesis to improve the oxygen and nutrition supplement, further contributing to the metastasis. Although the host immune cells attempt to eliminate cancer cells, these immune cells would be inactivated or differentiated under the regulation of TME. According to the situation of infiltrated immune cells, the tumor can be divided into a hot tumor (with abundant infiltrated immune cells) or a cold tumor (with limited infiltrated immune cells).^[^ ^57^ ^]^ Therefore, regulating the TME gradually becomes an attractive therapeutic method in tumor treatment.

In this section, we will systematically introduce the functionality of dendritic cells (DCs), tumor‐associated macrophages (TAMs), tumor‐associated fibroblasts (TAFs), endothelial cells (ECs), myeloid‐derived suppressor cells (MDSCs), and tumor‐associated T cells (Treg) in TME, as shown in **Table** **3**. We also briefly introduce how the other special characteristics, such as hypoxia, dense, and acid surroundings, influence the TME.

**Table 3:** The brief summary of various cells in the TME

Cell	Biomarkers	Functions in the cancer immunotherapy	NPs applied for regulating cells. Ref.
DCs	CD40, CD86, and CD103	Mediating anti‐tumor immune response, inducing the differentiation of effector memory T cells,	[65, 66, 67]
TAMs	CD206, CD163, and Arg1	Contributing to proliferation of tumor cells, extracellular matrix composition, and angiogenesis	[71, 72, 73]
TAFs	*α*‐SMA and FAP	Promoting tumor invasion and metastasis	[46, 79, 80]
ECs	CD 248, CD31, CD105, and CD146	Transporting immune cells, activating innate and adaptive immune responses, angiogenesis, and tumor metastasis	[90, 91, 92]
MDSCs	CD84, CD71, and CD86	Inhibiting the immune responses	[97, 98, 99]
Treg	Foxp3	Suppressing the self‐reactive T cell populations	[100‐102]

### 3.1 Dendritic Cells

Dendritic cells (DCs) are typical antigen‐presenting cells (APCs) of the immune system. DCs circulate in the body; when they capture the foreign antigens, they will be activated and present the antigens to the lymphocytes, such as T cells and B cells, to activate them.^[^ ^58^ ^]^ In addition, DCs participate in both innate immune response and adaptive immune response and become a bridge connecting the innate immune response and adaptive immune response.^[^ ^59^ ^]^ When a tumor occurs in the body, DCs infiltrate solid tumors and capture and process these antigens to a complex with major histocompatibility complex (MHC) molecules. DCs migrate to draining lymph nodes (LN) and present these antigens to CD4^+^ or CD8^+^ T cells; then, there will be two consequences. Either the T cells become quiescent and differentiate into an immune regulatory T cell, or T cells are activated with induced anti‐tumor capability; the last consequence is the desired effective anti‐tumor immune response. Although other APCs, such as macrophages, can contribute to the last consequence, DCs are considered the most proficient of all APC types. Moreover, there are three necessary compositions to activate the T cells. First, the DCs process the exogenous antigens from cancer cells into MHCI or MHCII molecules, presenting them to CD8^+^ T cells or CD4^+^ T cells, respectively. However, presenting the MHC molecules to T cells without any inflammatory signals cannot effectively activate the T cells.^[^ ^60^ ^]^ Under the stimulation from the tumor, DCs can express the various receptors that can detect danger signals, such as pathogen‐associated molecular patterns (PAMPs), toll‐like receptors (TLRs), NOD‐like receptors (NLRs), and C‐type lectins. Moreover, the ligands for these receptors are expressed in T cells. In addition, the expression of co‐stimulatory molecules, such as CD40^+^ and CD86^+^, is increased on DCs. Under the regulation of MHC molecules, co‐stimulatory molecules, and detected danger signals, T cells can be effectively activated. After activation, T cells need inflammatory cytokines, such as interleukin (IL)‐12 and IL‐15, to boost and sustain their effector status and enable them to keep expanding. DCs can provide these inflammatory cytokines, helping T cells in the activated status with proliferation. In clinical settings, the presence of DCs in the primary tumor is positively related to the survival rate of patients with pancreatic, lung, and breast cancers.^[^ ^61^, ^62^ ^]^

However, in the TME, some factors, such as mucins, gangliosides, neuropeptides, and nitric oxide, can shorten the lifespan of DCs. In addition, although DCs can capture some danger signals from the tumor, most solid tumors do not generate any danger signals to DCs without the influence of exogenous inflammatory signals. Moreover, cancer cells develop various strategies for inhibiting the DCs‐mediated anti‐tumor immune response. For example, cancer cells can activate the transcription factor Xbox binding protein (XBP), causing the endoplasmic reticulum stress in DCs, which disrupts the priming of T cells.^[^ ^63^ ^]^ Cancer cells can also activate the B‐catenin signaling pathway, hindering the recruitment of DCs in tumors. In TME, the IL‐10 secreted by the tumor‐associated macrophages can bind to the CD103^+^ DCs with the high expression of the IL‐10 receptor, which further causes the decreased secretion of IL‐12 in DCs. The downregulated concentration of IL‐12 will hinder CD8^+^ T cell proliferation and cytotoxicity. Furthermore, the IL‐10 can also induce the differentiation of monocytes to immunosuppressive M2 macrophages rather than inflammatory M1 macrophages or monocyte‐derived DCs.^[^ ^64^ ^]^

**Figure 3:**
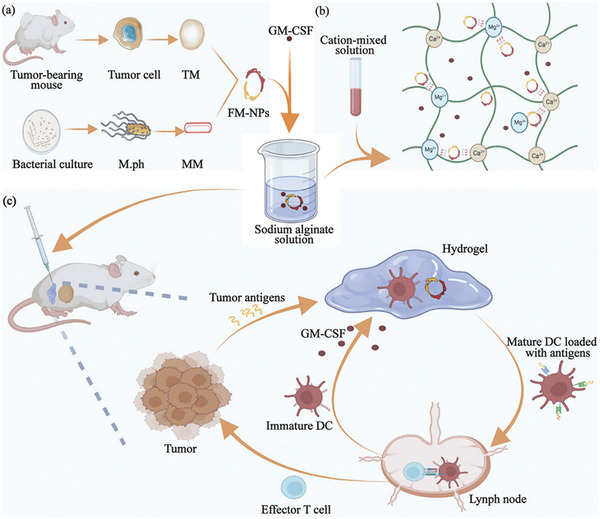
Schematic depiction of a) preparing the FM‐NPs, b) preparing the GM‐SCF and FM‐NPs contained alginate–cation hydrogels, and c) the hydrogel inducing the DCs maturation and activating the effector T cells to inhibit the tumor. Reproduced with permission.^[^ ^65^ ^]^ Copyright 2022, Wiley‐VCH GmbH.

To improve the potential DCs‐mediated anti‐tumor immune responses, the researcher developed various strategies to activate the DCs and boost the anti‐tumor immune responses. As shown in **Figure** **3**, Ke et al. fabricated bifunctional fusion membrane nanoparticles (FM‐NPs) composed of autologous tumor cell membranes (providing the tumor‐associated antigens) and Mycobacterium phlei membrane extracts (as an adjuvant to active the DCs). Then, the NPs were loaded into an injectable alginate hydrogel with granulocyte‐macrophage colony‐stimulating factor (GM‐CSF) that was used for recruiting DCs. Furthermore, the hydrogel could enhance the maturation of DCs in tumor‐draining lymph nodes and induce the differentiation of effector memory T cells, migrating to the TME and converting the cold tumor to the hot tumor.^[^ ^65^ ^]^

Zhang et al. prepared tumor‐permeated adenosine triphosphate (ATP)‐based NPs as the ATP is necessary for triggering the eat‐me signal in immunogenic cell death (ICD). Before arriving at the tumor, the NPs can protect ATP from degradation. Under the stimulation of MMP2 in TME, the NPs can release ATP that can act as an eat‐me signal for DCs maturation. Effective tumor inhibition was observed in the 4T1‐bearing mice treated with the NPs.^[^ ^66^ ^]^ Sun et al. fabricated hybrid intelligent DCs (iDCs) by coating the cell membrane of mature DCs on the photothermal agents‐loaded NPs. The iDCs could enter the lymph node and present the antigen to the T cells, followed by activating the T cells. In addition, the activating T cells could secret the heat shock proteins (HSPs) that could make tumor cells more sensitive to heat stress. Furthermore, the ICD was induced under mild photothermal therapy (42–45 °C), contributing to a synergistic antitumor effect.^[^ ^67^ ^]^

### 3.2 Tumor Associated Macrophages

Macrophages are highly plastic cells that can respond to and adapt the external stimulations. After stimulation of inflammatory factors, such as lipopolysaccharide (LPS), interferon (IFN)‐*γ*, or tumor necrosis factor (TNF)‐*α*, the macrophages can further differentiate into a pro‐inflammatory subtype M1 macrophages. On the one hand, M1 macrophages exhibit enhanced antigen‐presenting capability with the secretion of inflammatory cytokines, such as IL‐6, IL‐12, TNF‐*α*, IFN‐*γ*, and ROS. Usually, the M1 macrophages are identified by the expression of Nos2, Ciita, and IL12b. On the other hand, the macrophages can also differentiate into an anti‐inflammatory subtype, M2 macrophages, by inducing IL‐4, IL‐13, IL‐10, or glucocorticoids. The M2 macrophages can produce anti‐inflammatory cytokines, such as transforming growth factor (TGF)‐*β* and IL‐10, contributing to cell proliferation, angiogenesis, and tissue regeneration. The M2 macrophages are also denoted as tumor‐associated macrophages (TAMs) in TME, with the identified markers as Arg1, Chi3l3, and Retnla.^[^ ^68^, ^69^ ^]^

In the beginning, the M1 macrophages play anti‐tumor roles, but the generated oxidative stress and anti‐apoptotic responses contribute to carcinogenesis, leading to cellular survival and renewal. Under chronic inflammation in the tumor, the TME is progressively regulated by the M2 macrophages by the production of TGF‐*β*, regulating extracellular matrix composition and angiogenesis in tumor stroma. Moreover, the sustained released TGF‐*β* also contributes to the proliferation of tumor cells. Several signal pathways are involved in M1/M2 polarization: signal transducer and activator of transcription (STAT)1, nuclear factor kappa‐light‐chain‐enhancer of activated B cells (NF‐*κ*B), and interferon regulatory factor 5 (IRF5) are associated with M1 polarization, whereas STAT6, MYC, IRF4, kruppel‐like factor 4 (KLF4), and peroxisome proliferator‐activated receptor gamma (PPAR*γ*) are involved in M2 polarization. Although various non‐malignant stromal cells are present at the complex TME, TAMs are one of the most represented populations in the TME as an indicator of tumor invasion, angiogenesis, and metastasis. Therefore, TAMs are becoming an attractive target for tumor therapy. Recently, research on TAMs has focused on the following aspects, reprogramming the M2 macrophages to M1 macrophages, depleting TAMs, and suppressing the recruitment of macrophages.^[^ ^70^ ^]^

Reprograming TAMs to pro‐inflammatory subtypes is an approach to cancer immunotherapy. Figueiredo et al. conjugated an M2‐macrophages targeted peptide (mUNO) with resiquimod‐loaded lignin nanoparticles, which aimed to reverse the anti‐inflammatory M2 macrophages to the pro‐inflammatory M1 macrophages. In the 4T1 tumor‐bearing mice model, the NPs exhibited homing capability to the tumor. In addition, the NPs inhibited tumor growth with satisfactory biocompatibility compared to the commercial chemotherapy drug (vinblastine). Moreover, the NPs could also activate the TME by increasing the ratio of M1/M2 macrophages and the infiltration of cytotoxic T cells.^[^ ^71^ ^]^

**Figure 4:**
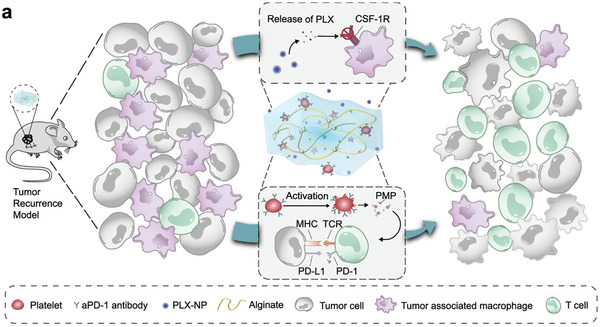
Schematic illustration of the mechanism of tumor immune suppressive microenvironment modulation capability of PLX‐NPs and P‐aPD‐1 loaded alginate‐based hydrogel in the tumor recurrence model. Reproduced with permission.^[^ ^72^ ^]^ Copyright 2022, Springer Nature.

Depleting TAMs is another strategy for cancer immunotherapy. As shown in **Figure** **4**, Li et al. co‐loaded pexidartinib (PLX)‐loaded dextran nanoparticles (PLX‐NPs) and anti‐programmed death‐1 (PD‐1) antibody‐conjugated platelets (P‐aPD‐1) into an alginate hydrogel. In the recurrent B16F10 tumor model, hydrogels were implanted in the surgical tumor cavity. The decreased density of TAMs and increased density of CD8^+^ T cells were observed in the tumor tissues. Furthermore, the capabilities of depleting TAMs, promoting the infiltration of CD8^+^ T cell and inhibiting lung metastasis, were observed in mice (CT26 and B16F10 tumor recurrence models and metastatic 4T1 breast tumor recurrence model) treated with hydrogel.^[^ ^72^ ^]^

Utilizing M2 macrophages to transport the NPs could be a promising strategy in cancer treatment. Although the TME was complex, Lin et al. proved that TAMs actively migrated toward NPs extravasated from the vessels, engulfing and redistributing them in the tumor stroma. The TAMs could deliver NPs two to five times more profound than the passive diffusion; the NPs were first involved in the uptake of TAMs and then redistribution. The degree of NPs delivery via TAMs and diffusion was size‐dependent. NPs with smaller sizes (15, 50, and 100 nm in this experiment) tended to diffuse for transport in the TME.^[^ ^73^ ^]^ This study provides another possibility to reconsider the TAMs in the TME.

### 3.3 Tumor‐Associated Fibroblasts

Besides the TAMs, another dominant population in TME is the tumor‐associated fibroblasts (TAFs). Although research supports that fibroblasts originate from primitive mesenchymal cells, TAFs originate from activated fibroblasts in local tissues.^[^ ^54^ ^]^ Cancer cells can induce the normal fibroblasts to differentiate to TAFs through exosome‐mediated transforming growth factor beta (TGF‐*β*) transmission and SMAD signaling pathways.^[^ ^74^ ^]^ In addition, the cancer cell can also induce the differentiation of endothelial cells into TAFs through the endothelial–mesenchymal transition (EndMT).^[^ ^75^ ^]^ Moreover, the epithelial cells can be potentially differentiated into TAFs by epithelial–mesenchymal transition (EMT).^[^ ^76^ ^]^ Consequently, TAFs can secret the cytokines to promote the EMT of tumor cells, which further promote tumor invasion and metastasis. In addition, the TGF‐*β* can differentiate bone marrow‐derived mesenchymal stem cells into TAFs by activating the JAK/STAT3 signaling pathway. Besides the diverse origins of TAFs, TAFs also exhibit various biomarkers but there is no one specific biomarker for the TAFs. Generally, the biomarker of TAFs can be divided into cytokines (IL‐6, IL‐32, C‐X‐C motif ligand 14, and C‐C motif ligand 2/CCL8), growth factors (TGF‐*β*1, hepatocyte growth factor, and epidermal growth factor), receptors (integrin *α*11/Platelet‐derived growth factor *β*, fibroblast activation protein, fibroblast growth factor receptors, discoidin domain receptor 2, and vascular cell adhesion protein 1), cell‐penetrating peptides, and cytoskeleton components (*α*‐smooth muscle actin, netrin‐1, and vimentin).^[^ ^77^ ^]^

There are several potential mechanisms in activating the TAFs. For example, IL‐1 promotes the activation of TAFs by the NF‐*κ*B, and IL‐6 activates the TAFs by the JAK‐STAT signaling pathway. The stromal stiffness and external mechanical force also activate the TAFs. Moreover, the activated TAFs can maintain the activated status and stimulate the cancer cells to support their activation further.^[^ ^78^ ^]^ After activation, TAFs will remodel the ECM, rebuild the tumor‐associated vessels, regulate the inflammation, and modify the stromal stiffness, which could influence tumor initiation, progression, and therapeutic resistance. Therefore, increasing numbers of researchers regard TAFs as a potential target for anti‐tumor therapy.

Liu et al. designed a multi‐functional NPs platform that could transform the activated TAFs to a quiescent state by down‐regulating the expression of *α*‐SMA, fibroblast‐activated protein (FAP), and fatty acid synthetase (FASN). Consequently, the intercellular pressure was reduced, promoting the penetration of the NPs into the deep tumor.^[^ ^46^ ^]^ Zheng et al. self‐assembled the PSN38 (prodrug of SN38) with triptolide‐naphthalene sulfonamide (TPL‐nsa) to prepare the NPs. The NPs could reverse the TAF‐induced gastric cancer cell proliferation, migration, and chemotherapy resistance by transforming the activated TAFs into a quiescent state, which could be attributed to the inhibited expression of FAP and *α*‐SMA in TAFs, and also the inhibition of the NF‐*κ*B and TGF‐*β* signaling pathways.^[^ ^79^ ^]^

As the TAFs support the metabolism of cancer cells, blocking this process could be a promising approach to treating cancer. Zang et al. fused the 4T1 cancer cell membrane with the activated NIH373 fibroblast membrane to prepare the hybrid cell membrane, aiming to target cancer cells and TAFs homologously. Furthermore, the PTX and PFK15 co‐loaded solid lipid nanoparticles were coated with the hybrid cell membranes. Herein, the PTX was used as a chemotherapeutic drug, and PFK15 was used to block glycolysis, hinder energy production, and cut off the feeding of glycolytic metabolites from TAFs to cancer cells. After blocking the glycolysis, the production of lactate was decreased, which benefited downregulating the percentage of Treg, reprogramming M2 to M1 macrophages, and increasing the percentage of both CD8^+^ and CD4^+^ T cells.^[^ ^80^ ^]^

### 3.4 Endothelial Cells

The constant endothelial cells (ECs) construct the endothelium that can organize the growth of connective tissues and form the surrounding blood vessels. In addition, the endothelium delivers nutrition, maintains metabolic homeostasis, transports immune cells, and activates innate and adaptive immune responses and angiogenesis.^[^ ^81^ ^]^ Moreover, ECs can sense and respond to changes in their surroundings. In the TME, some signals, such as hypoxia and chronic growth factor, can induce the dysfunction of ECs, and the dysfunctional ECs further contribute to cancer progression.^[^ ^82^ ^]^ During the progress of the tumor, there are two phases, the avascular growth phase and the vascular growth phase. Initially, the tumor is noninvasive, with a size lower than 1–2 mm. Then, the tumor tends to be invasive, and the invasion is angiogenesis‐dependent because the tumor also needs nutrition supplements and waste disposal. Furthermore, angiogenesis is initiated by ECs activation. In the clinic, there are several approaches targeting angiogenesis and ECs for anti‐tumor therapy: 1) binding the circulating vascular endothelial growth factor (VEGF) by the neutralizing monoclonal antibody, 2) blocking the tyrosine kinase activity of VEGFRs by small molecules inhibitors, and 3) binding the VEGFR‐2 by the monoclonal antibody.^[^ ^83^ ^]^

Some tumor endothelial markers can be applied to identify the ECs in TME. For example, endosialin (CD 248), the expression of CD248 in the tumor endothelium, is dramatically higher (5‐ to 20‐fold) than that in the endothelium of normal tissues.^[^ ^84^ ^]^ G‐protein‐coupled receptor 124 (GPR124) regulates the proliferation of human umbilical vein endothelial cells (HUVEC).^[^ ^85^ ^]^ Morevoer, the activation of GPR124 is associated with cell‐to‐cell contacts during capillary morphogenesis.^[^ ^85^ ^]^ In the TME, the GPR124 is highly expressed in the ECs. Plexin domain containing 1 (PLXDC1) also contributes to capillary morphogenesis, and PLXDC1 is highly expressed in various cancers, such as colorectal, lung, pancreas, breast, brain, and osteogenic cancer.^[^ ^86^, ^87^ ^]^ Anthrax toxin receptor (ATR) participates in cell‐to‐cell and cell‐to‐extracellular matrix communications, helping the adhesion and migration of cells.^[^ ^88^ ^]^ The high expression of ATR is proven in various cancers, such as breast, gallbladder, and colorectal cancer; the high expression of ATR is usually associated with shorter survival periods.^[^ ^89^ ^]^

As endogenous bioelectricity can intervene in vessel formation, Li et al. prepared polarized barium titanate NPs with high mechano‐electrical conversion performance. The NPs could generate pulsed open‐circuit voltage under low‐intensity pulsed ultrasound, which further disturbed the intracellular calcium ion gradient, inhibited angiogenesis‐related eNOS/NO pathway, and inhibited endothelial cell migration and differentiation. Consequently, the tumor vasculature was normalized with enhanced blood perfusion, reduced vascular leakage, and restored local oxygenation. Together with the DOX, the therapeutic effects were raised 1.8 times compared to only DOX.^[^ ^90^ ^]^ Wang et al. fabricated superparamagnetic NPs modified with oleic acid, erlotinib, and bevacizumab. The expressions of CD31 and *α*‐SMA were monitored 0, 1‐, 3‐, 5‐, and 7‐days post‐injection of NPs. Interestingly, the normalization of tumor vasculature was dynamically changed. Three days post‐injection, the tumor blood vessels began to normalize; this phenomenon was further enhanced on 5‐and 7‐days post‐injection.^[^ ^91^ ^]^

Unlike other solid cancers, angiogenesis could be beneficial in treating bone cancer because the defected bone should be generated after the surgery. Pang et al. synthesized Cu and Mn‐doped borosilicate nanoparticles (BSNs). There were three functionalities in these NPs and cancer cells were inhibited by the hydroxyl radicals produced by the Fenton‐like reactions of Cu^2+^ and Mn^3+^ ions; bone regeneration was also promoted by the Cu^2+^ and Mn^3+^ ions; in addition, the BSNs can also promote the proliferation of ECs (EaHy926), followed by the enhanced angiogenesis.^[^ ^92^ ^]^

### 3.5 Myeloid‐Derived Suppressor Cells

Pathologically activated neutrophils and monocytes with potential immunosuppressive capability are denoted as myeloid‐derived suppressor cells (MDSCs).^[^ ^93^ ^]^ In addition, MDSCs are associated with unsatisfactory outcomes in cancer therapy. There are two major populations of MDSCs, granulocytic/polymorphonuclear MDSCs (PMN‐MDSCs) and monocytic MDSCs (M‐MDSCs), which are respectively classified according to their origin cell lineages. In the TME, due to prolonged inflammatory stimulation, such as GM‐CSF, IL‐6, and IL‐1*β*, the myeloid cells expand and are constantly activated, which is related to the emergence of MDSCs. In addition, the development of MDSCs is regulated by the following molecular pathways, such as STAT1, STAT3, STAT6, and NF‐*κ*B.^[^ ^94^ ^]^

The primary function of MDSCs is inhibiting the immune responses, such as T cells, B cells, and NK cells mediated immune responses, by upregulating the STAT3, inducing the endoplasmic reticulum (ER) stress, and expressing arginase 1 and S100A8/A. However, PMN‐MDSCs and M‐MDSCs prefer to use different approaches to mediating immunosuppression. PMN‐MDSCs inhibit the immune responses of arginase 1, peroxynitrite, and prostaglandin E2 (PGE2). Meanwhile, M‐MDSCs induce immunosuppression by nitric oxide (NO), IL‐10, and TGF‐*β*. Studies also revealed markers to identify the MDSCs, such as CD84, CD71, and CD86.^[^ ^95^, ^96^ ^]^ The MDSCs can also recruit regulatory T cells to inhibit the immune responses further and induce angiogenesis to support tumor invasion and metastasis. Therefore, MDSCs are also a potential target for cancer immunotherapy.

Liu et al. fabricated complex liposome systems consisting of colorectal cancer cell membranes, short hair‐pinned RNA (shRNA) against plasmacytoma variant translocation 1 (Pvt1), 1,2‐Dioleoyl‐3‐trimethylammonium propane (DOTAP), and oxaliplatin. The complex liposomes can induce the ShPvt1‐CM‐D‐mediated Pvt1 knockdown and enhance oxaliplatin‐induced ICD; then, the released ICD could be as other antigens to trigger the maturation of DCs; ShPvt1 can knockdown the expression of Pvt1 in PMN‐MDSCs, ameliorating the PMN‐MDSCs‐mediated immunosuppression.^[^ ^97^ ^]^

**Figure 5:**
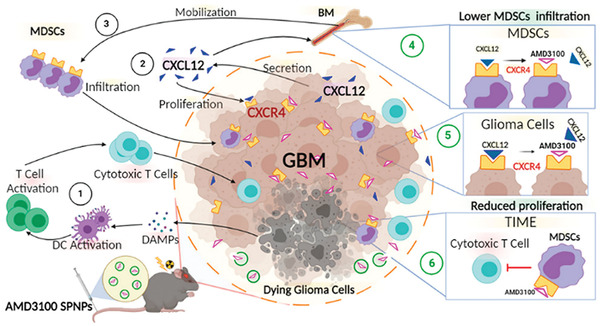
Potential mechanism of AMD3100 SPNPs in an aggressive intracranial glioblastoma multiforme (GBM) model. The blocking CXCR4 can reduce infiltration of CXCR4^+^ M‐MDSCs to the GBM TME in vivo. In addition, blocking CXCR4 sensitized GBM cells to radiation‐induced ICD triggers an anti‐GBM adaptive immune response. With the potent ICD induction and reprogrammed immunosuppressive microenvironment, SPNPs elicit antigen presentation, immune priming, and GBM‐specific immunological T‐cell‐mediated immunity. Reproduced with permission.^[^ ^98^ ^]^ Copyright 2022, American Chemical Society.

As shown in **Figure** **5**, Alghamri et al. developed NPs modified with the transcytotic peptide iRGD (AMD3100‐SPNPs) to target the C–X–C motif chemokine ligand‐12/C–X–C motif chemokine receptor‐4 (CXCL12/CXCR4) pathway. Consequently, the NPs block CXCL12/CXCR4 signaling, leading to the inhibited glioblastoma proliferation and reduced infiltration of CXCR4^+^ M‐MDSCs into the TME, restoring the integrity of the blood–brain barrier (BBB), and induced ICD.^[^ ^98^ ^]^

As the Fusobacterium nucleatum contributes to the increased MDSCs in colorectal cancer, Dong et al. self‐assembled a specifically Fn‐binding M13 phage with silver nanoparticles through the electrostatic interaction to precisely clean the Fn and remodel the TME. As a result, the clearance of Fn and decreased population of MDSCs were observed on the colorectal cancer site, followed by the activation of APCs. Together with immune checkpoint inhibitors (*α*‐PD1) or chemotherapeutics (FOLFIRI), NPs can significantly prolong the overall mice survival in the orthotopic colorectal cancer model.^[^ ^99^ ^]^

### 3.6 Regulatory T Cells

Regulatory T cells (Treg) are a subtype of CD4^+^ T cells; the expression of Forkhead box protein 3 (Foxp3) can be used to distinguish Treg from other immune cells. Treg plays an essential role in maintaining self‐tolerance and homeostasis by suppressing the self‐reactive T cell populations. The chemokines, such as CXCL12, CCL20, CCL5, CCL28, and CCL2/22, guide the Treg homing to the tumor. Then, Treg can crosstalk with other cells in TME, such as MDSCs, TAMs, DCs, TAFs, ECs, and tumor cells, which could inhibit the maturation of APCs with the secretion of inhibitory cytokines. Therefore, attempts to deplete Treg to regulate the TME have been studied.

The deletion or mutation of tumor suppressor genes, such as the gene‐encoding phosphatase and tensin homolog deleted on chromosome 10 (PTEN) in cancer cells may associate with the immunosuppressive TME and poor response or resistance to immune checkpoint blockade (ICB) therapy. Lin et al. delivered the mRNA by polymeric NPs to induce the expression of PTEN in Pten‐mutated melanoma cells and Pten‐null prostate cancer cells, which activated the TME by promoting CD8^+^ T cell infiltration, enhancing the secretion of proinflammatory cytokines, reducing the Treg.^[^ ^100^ ^]^ Song et al. prepared IPI‐549 and PTX co‐loaded albumin NPs. Together with the anti‐PD1, the NPs remodeled the TME in both tumor and lymph nodes by reprogramming M2 to M1 macrophages, improving drug concentration, increasing CD4^+^ and CD8^+^ T cells, B cells, and dendritic cells, decreasing regulatory T cells, and preventing T cell exhaustion.^[^ ^101^ ^]^ Peng et al. combined the irreversible electroporation with GSH‐responsive degradable NPs loading with a TGF‐*β*1 inhibitor (SB525334) in treating pancreatic cancer. The irreversible electroporation could induce a substantial infiltration of neutrophils into pancreatic tumors. Then, the local inhibition of TGF‐*β*1 could promote the neutrophils polarizing to an antitumor phenotype, enhance the CD8^+^ T cell infiltration, and deplete Treg.^[^ ^102^ ^]^

### 3.7 Other Characteristics in TME

Besides the above‐mentioned specific cell compositions, the TME is also famous for the acid pH, hypoxia environment, and dense extracellular matrix. Therefore, we will briefly introduce the causes of these special characteristics in TME and how these characteristics influence tumor progress and treatments. As a major population in the TME, TAFs can secrete collagen types I and III, fibronectin, proteoglycans, and glycosaminoglycans, which increase mechanical pressure in the extracellular matrix and contribute to the formation of dense TME. Consequently, the dense TME limits the supply of oxygen and promotes tumor invasion, angiogenesis, and metastasis.^[^ ^103^ ^]^ Furthermore, due to the limited oxygen and blood supply and the uncontrolled proliferation, the TME is becoming hypoxic from the average oxygen level of 2–9% to less than 2%. Then, these cancer cells will adapt to the hypoxia environment, causing changes in gene expression and subsequent proteomic, which leads to more aggressive and therapeutically resistant tumor phenotypes.^[^ ^104^ ^]^ Uncontrolled proliferation, abnormal metabolism, and insufficient penetration also contribute to the pH changes in the TME. The uncontrolled proliferation needs the support from glycolysis, which would release abundant H^+^. Meanwhile, insufficient penetration limits both oxygen delivery and H^+^ removal in tumors together with the elevated oxidative metabolism, which further contributes to the acid TME. In general, the pH value of most tumor tissues is in the range of 6.4–7; although some pH of the human tumor tissues can decrease to 5.6. After long‐term exposure to the acid TME, these cancer cells are fundamentally changed in their genotype and phenotype, which could increase cancer cell fitness and aggressiveness.^[^ ^105^ ^]^

### 3.8 Summary

In the solid tumor, the TME consists of various cells, such as DCs, TAMs, TAFs, ECs, MDSCs, and Tregs. Besides these cell compositions, the TME is also famous for the acid pH, hypoxia environment, and dense extracellular matrix. Together, these elements communicate with each other, contributing to the tumor progress, metastasis, drug resistance, and cold immune environment. Interestingly, the TME is not unchanged but the TME is dynamic and changeable. The TME can be changed by regulating these tumor‐associated cells or cytokines, which provides potential therapeutic approaches to cancer immunotherapies.

However, there are still some challenges in regulating the TME. For example, the M2 macrophages contribute to carrying NPs to the deep tumor, but the M2 macrophages also participate in suppressive TMEs. Therefore, the relationship among TME, NPs, and therapeutic effects should be carefully considered with an optimized balance and therapeutic outcomes.

## 4 Nanoparticle on Regulating Tumor Microenvironment and Cancer Immunotherapy

**Scheme 3:**
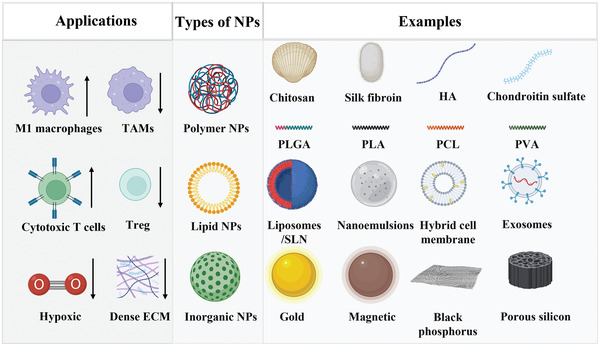
The polymer NPs are divided into natural (chitosan, silk fibroin, HA, and chondroitin sulfate) and synthetic (PLGA, PLA, PCL, and PVA) NPs, according to the source of the polymer. The lipid NPs commonly consist of liposomes, SLN, nanoemulsions, hybrid cell membrane, and exosome NPs. The inorganic NPs are gold, magnetic, black phosphorus, and porous silicon NPs. These NPs can be applied in regulating TME and cancer treatments by increasing the ratio of M1 macrophages and cytotoxic T cells, decreasing the ratio of TAMs and Treg, and reducing the hypoxic and dense environments. Created with BioRender.com.

In most solid tumors, the complex TME, such as dense ECM, high concentration of GSH, abnormal angiogenesis and vascular networks, hypoxia, limited immune cells infiltration, and acid pH, contribute to the tumor progress and metastasis with close interactions. As a modern weapon, NPs have unique advantages, such as tailored physicochemical properties, active or/and passive targeting capabilities, photothermal or/and photochemical effects, responsive drug releases, and so on. These advantages make NPs a promising candidate for regulating the TME, as shown in **Scheme** **3**.

### 4.1 Polymer‐Based NPs

Generally, polymer‐based NPs were divided into three types: natural polymer‐based NPs, synthetic polymer‐based NPs, and biosynthetic polymer‐based NPs, according to the polymer source. Currently, the commonly used natural polymers are chitosan, silk fibroin, hyaluronic acid (HA), and chondroitin sulfate.^[^ ^106^ ^]^

Chitosan is biocompatible and biodegradable and tends to bind to negatively‐charged proteins or plasmid DNA through electrostatic interaction. In addition, the abundant amino and hydroxyl groups make chitosan be chemically modified for various purposes. Maryam et al. modified chitosan with lactate to increase the siRNA loading efficacy; then, the NPs blocked the expression of cytotoxic T‐lymphocyte antigen 4 (CTLA‐4) on infiltrating T cells. Consequently, the cytotoxicity of T cells was increased with an increased concentration of granzyme B, IFN‐*γ*, the decreased concentration of IL‐10, and the constant concentration of IL‐17.^[^ ^107^ ^]^ Shen et al. utilized the interaction between iodinated indocyanine green (ICG) and chitosan to self‐assemble the NPs, followed by coating with MnO_2_ through the electrostatic interaction. Under near‐infrared (NIR) irradiation, the NPs generated heat and oxygen and consumed the GSH, triggering acute immune responses. Moreover, the generated heat accelerated the blood flow, relieving the hypoxia and regulating the TME.^[^ ^108^ ^]^

Silk fibroin is usually derived from domesticated silkworm (Bombyx mori) cocoon as a protein with a high molecule. Silk fibroin is negatively charged with stimuli‐responsive self‐assembly behavior. Yu et al. prepared a metal–organic framework consisting of Fe^3+^ and 4,4,4,4‐(porphine‐5,10,15,20‐tetrayl) tetrakis (benzoic acid) (TCPP), followed by coating with silk fibroin and tirapazamine. After uptake by the cancer cells, the NPs increased the ROS generation and consumed the GSH by the Fenton reactions. Then, the enhanced ICD was induced by the increased intracellular ROS and the stimulation of silk fibroin. In addition, the silk fibroin contributed to reprogramming M2 to M1 macrophages, regulating the TME.^[^ ^109^ ^]^

HA, as a polysaccharide, can specifically target the CD44 surficial receptor on cancer cells, and HA is usually considered to have a short biological half‐life. Du et al. loaded curcumin into HMnO_2_ NPs, followed by coating with HA and Nrp‐1 binding monoclonal antibodies (mAb). Due to the targeting capability of HA, the NPs actively achieved at the tumor and degraded in the cancer cell, leading to the apoptosis of cancer cells with enhanced immunogenicity. Meanwhile, the mAb could block the Treg with relieved immunosuppression.^[^ ^110^ ^]^ Similarly, Sun et al. also utilized the targeting capability of HA to guide the HA‐functionalized magnetite nanoparticles arriving at the tumor site. When the NPs arrived at the tumor, the high intratumoral concentration of HA could directly recruit lymphocytes and induce the secretion of chemokines, such as CXCL9, CXCL10, CXCL11, and CCL2, through the cascading amplification effects. Then, the cold TME was turned into hot TME by reprograming the infiltrating macrophages to an anticancer M1 phenotype in 4T1‐bearing mice.^[^ ^111^ ^]^ Singh et al. synthesized the gemcitabine and imiquimod conjugated HA by the standard N‐(3‐dimethylaminopropyl)‐N′‐ethylcarbodiimide/N‐hydroxysuccinimide (EDC/NHS) chemistry. When a 4T1 cancer cell sphere was treated with the HA NPs, infiltration of THP‐1 monocytes occurred at the treated site. 4T1‐bearing mice treated with HA NPs exhibited the increasing activation of CD11b^+^ immune cells in the blood.^[^ ^112^ ^]^

Chondroitin sulfate, as a glycosaminoglycan, is widely distributed in the cell membranes and ECM in the form of proteoglycans (denoted as chondroitin sulfate proteoglycans, CSPGs). CSPGs participate in various physiological processes, such as cytokinesis and morphogenesis. Chen et al. genetically modified T cells with CSPG‐4 to prepare the chimeric antigen receptor (CAR)‐redirected T lymphocytes (CAR T cells). Together with the mild hyperthermia caused by the ICG‐loaded PLGA NPs, increased blood perfusion, monocytes, and dendritic cells were observed in tumor‐bearing mice with raised chemokines such as CCL5, CCL11, CXCL1, CCL2, CCL3, and CCL4.^[^ ^113^ ^]^ Li et al. co‐delivered chlorin e6 (Ce6) and retinoic acid (RA) into chondroitin sulfate‐based NPs. The NPs could reduce the photodynamic therapy (PDT)‐mediated immunosuppression by disturbing the apparatus of Golgi and blocking the secretion of immunosuppressive cytokines, which could be combination therapy with the PDT.^[^ ^114^ ^]^

**Figure 6:**
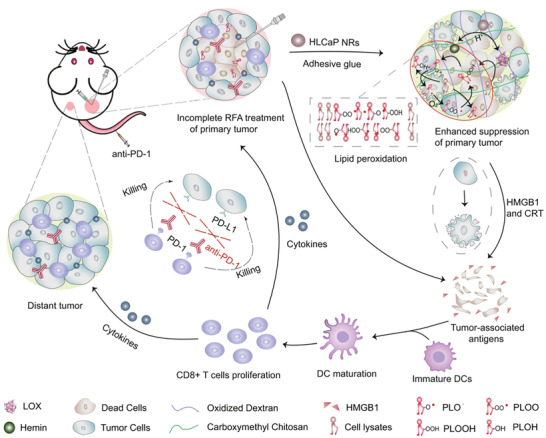
Under the acid TME, HLCaP NRs gradually released lipoxidase (LOX) hemin, and synergistically caused continuous lipid peroxidation from these polyunsaturated fatty acids (PUFA) containing phospholipids inside the tumor debris to trigger ferroptosis of residual tumor cells. Meanwhile, the released high mobility group box 1 (HMGB1) molecules from these ferroptotic cancer cells would recruit immature DCs to the residual tumor site and prime specific antitumor immune response featured in increased infiltration of effector T cells and secretion of effector cytokines, to further inhibit the growth of both residual tumors and metastatic (distant) tumors, especially in the combinational use of anti‐PD‐1 immunotherapy. Reproduced with permission.^[^ ^115^ ^]^ Copyright 2021, Springer Nature.

There are various synthetic polymers, such as PLGA, polylactic acid (PLA), polycaprolactone (PCL), and poly(vinyl alcohol) (PVA), applied for regulating TME. PLGA is synthesized by the ring‐opening co‐polymerization of cyclic dimers (1,4‐dioxane‐2,5‐diones) of glycolic acid and lactic acid. Owing to biocompatibility and biodegradability, the PLGA is a Food and Drug Administration (FDA)‐approved synthetic polymer, which is widely applied for medical purposes. As shown in **Figure** **6**, Yang et al. prepared hemin and lipoxidase co‐loaded CaCO_3_‐encapsulated PLGA NPs (HLCaP NRs). Due to the existence of CaCO_3_, the NPs can pH‐responsively induce the cytotoxic lipid radicals with these polyunsaturated fatty acids existing in cancer cell lysates, thereby inducing ICD. Together with anti‐PD‐l immunotherapy or radiofrequency ablation, the NPs can effectively eliminate primary residual tumors and even inhibit the growth of distant metastatic tumors.^[^ ^115^ ^]^

Koerner et al. loaded double‐stranded RNA adjuvant (Riboxxim) and ovalbumin (OVA) into PLGA NPs used as a cancer vaccine. The NPs vaccine triggered the maturation of DC and improved the cross‐presentation of CD8^+^ T cells. In addition, combined with anti‐CTLA‐4 immunotherapy, personalized tumor‐specific CD8^+^ T cells responses might be triggered in patients who do not respond to immune checkpoint blockade alone.^[^ ^116^ ^]^ Taking advantage of the solid properties of PLGA NPs, Chen et al. coated fused hybrid cell membranes (outer membrane vesicles of Salmonella and cell membranes of melanoma cells) on the ICG‐PLGA NPs. After irradiation with NIR, the NPs converted irradiation to heat, inducing the ICD and generating supplementary tumor‐associated antigens in the immune systems. The therapeutic efficacy of NPs was proved in both the B16F10 and 4T1 bearing mice with the increased percentage of CD3^+^ CD8^+^ CD107a T cells and CD3^+^ CD8^+^ CD44^+^ CD62L^−^ T cells.^[^ ^117^ ^]^

PLA can be systemized from nontoxic renewable feedstock with biocompatibility, biodegradability, and hydrophobicity, widely explored as drug delivery carriers, scaffolds, and wound dressing. Xu et al. assembled the PLA‐polyethylenimine (PEI) as the core structure that adsorbed the CpG and protein antigens, then modified it with a mannan shell. The modification of mannan enhanced the accumulation of NPs in lymph node draining and promoted the recruitment of CD8^+^ T cells and DCs. In addition, the inner core could increase the cross‐presentation between DCs and CD8^+^ T cells.^[^ ^118^ ^]^ Similarly, Hou et al. designed PTX‐loaded PLA‐PEI NPs modified with the HA‐inulin, aiming to deliver drugs to the colon by oral administration. Oral administration of NPs exhibited enhanced therapeutic effects compared to the intravenous injection of NPs in the orthotopic CT26‐bearing mice models by inducing the apoptosis of tumor cells.^[^ ^119^ ^]^

PCL is synthesized by ring‐opening polymerization of *ε*‐caprolactone under the catalyst of stannous octoate. PCL is also biodegradable with a low melting point of ≈60 °C, which makes it suitable for 3D printing. As for the application in tumor treatment, Wang et al. conjugated hemoglobin (Hb) with PCL to assemble the biomimetic nano red blood cell NPs loading with DOX. After binding to endogenous plasma haptoglobin (Hp), the NPs can specifically kill the M2 macrophages. In addition, the oxygen can be generated by the Hb on NPs, alleviating the tumor hypoxia and decreasing the recruitment of M2 macrophages. Consequently, the NPs can further decrease the expression of PD‐L1, and the secretion of IL‐10 and TGF‐*β*, with an increased percentage of CD8^+^ T cells in the 4T1‐bearing mice.^[^ ^120^ ^]^ Su et al. modified PCL with PEG, Ce6, and PBEMA to design a star‐shaped polymer that can assemble to NPs loading with quinone methide. The NPs can trigger the H_2_O_2_‐induced quinone methide release for GSH depletion, followed by ROS elevation and ICD generation.^[^ ^121^ ^]^

PVA is a water‐soluble polymer with satisfactory biocompatibility, low protein adhesion, and low toxicity. PVA is widely used for medical purposes, such as cartilage replacements, contact lenses, eye drops, and vascular stents. Regarding tumor treatments, Xu et al. stabilized polypyrrole (PPy) with PVA, loading with iron phosphate and decorating with glucose oxidase (GO*x*). In the TME, the NPs induced a cascade catalytic process. The GO*x*‐mediated glucose depletion generated abundant gluconic acid and H_2_O_2_, promoting the release of iron ions and triggering the Fenton reaction. Under apoptosis–ferroptosis, the tumor was inhibited with the regulated TME.^[^ ^122^ ^]^ Similarly, Mngadi et al. first fabricated ferrite NPs, then modified the NPs with PVA, followed by loading with 5‐fluorouracil (5‐FU) with 68% encapsulating efficiency. The NPs inhibited 65% of MCF‐7 and HeLa cancer cell proliferation.^[^ ^123^ ^]^ The presence of BBB limited transporting drugs to brain tumors; D. Mascolo et al. combined PLGA mesh laid over a PVA layer to prepare an implant. When the PVA layer dissolved, the PLGA mesh matched to the resected tumor cavity with the local release of DOX and diclofenac molecules to the tumor. In the orthotopic brain cancer models, a single implant carrying 0.75 mg kg^−1^ of DOX and diclofenac inhibited the tumor recrudesce for 8 months.^[^ ^124^ ^]^

### 4.2 Lipid‐Based nNPs

Lipid‐based NPs typically consist of a phospholipid, an ionizable lipid, cholesterol, and a PEGylated lipid. The first reported lipid‐based nanoparticle was liposomes, first described in 1961 by the British hematologist Alec D Bangham. After over 50 years of development, various lipid‐based NPs, such as liposomes, solid lipid NPs (SLN), nanoemulsions, exosomes, and cell membranes, were designed for medical purposes.^[^ ^125^ ^]^ Compared to polymer‐based NPs, lipid‐based nNPs have a more similar structure to cells, especially liposomes; the lipid‐based NPs are biocompatible and easily tailored for various applications, such as drug delivery and diagnosis.

Liposomes, as commercial NPs, are characterized by a spherical shape with a lipid bilayer. The surficial charge of liposomes can be easily changed by using different ionizable lipids. The common cationic lipids are 1,2‐di‐O‐octadecenyl‐3‐trimethylammonium propane (DOTMA), *N*‐[1‐(2,3‐dioleoyloxy) propyl]‐*N*, *N*, *N*‐trimethylammonium (DOTAP), and DLin‐MC3‐DMA. These cationic liposomes are widely applied as nucleic acid carriers for transfection and tumor treatments. Wang et al. prepared size and charger dual changeable gemcitabine prodrug‐loaded liposomes. Under the irritation of ultrasound, liquid–gas phase transition occurred in perfluoropentane, causing the size increase in liposomes from NPs to microparticles. With the prolonged duration of the ultrasound, liposomes transformed from microparticles to NPs, extravasating from blood vessels into the tumor periphery. Under the acid TME, the dimethyl maleic amides were hydrolyzed, triggering cationization‐initiated transcytosis. Satisfactory NPs penetration could be observed in the U251 glioma‐bearing mice with inhibited tumor growth.^[^ ^126^ ^]^ Jian et al. used liposomes co‐loaded zoledronic acid, IR780, and manganese dioxide (MnO_2_); then, the liposomes were further modified with LyP‐1 peptide. Triggered by the H_2_O_2_ in TME, the MnO_2_ generated O_2_ bubbles, degrading the liposomal membrane and releasing the zoledronic acid. The zoledronic acid could be selectively phagocytosed by TAMs, reprogramming M2 to M1 macrophages. The tumor cell could internalize the LyP‐1 peptide‐labeled fragments containing IR780. After the NIR irradiation, the tumor cell generated abundant ROS, causing mild immune activation.^[^ ^127^ ^]^

Different from the lipid bilayer of liposomes, SLN consists of solid fats and surfactants. The SLN can protect drugs in acidic pH with long shelf life, high cell uptake, biocompatibility, and biodegradability. Banerjee et al. designed a PTX‐loaded SLN modified with Tyr‐3‐octreotide (PSM) to treat melanoma with highly expressed somatostatin receptors (SSTRs). In the B16F10‐bearing mice, the NPs caused calreticulin exposure, leading to the increased infiltration of CD8^+^ T cells and induced ICD.^[^ ^128^ ^]^ Kim et al. used SLN to load docetaxel (DTX) to boost the intrinsic tumor‐fighting immune capacity, followed by coating with an anionic polymer conjugated with glycocholic acid. After oral administration, the NPs were actively absorbed in the distal ileum, mediated by interactions with the apical sodium bile acid transporter. The release of DTX could be sustained for up to 24 h in plasma without damaging the immune system. Moreover, an increased population of cytotoxic T cells and decreased population of TAMs and Treg were also observed in B16F10‐bearing mice.^[^ ^129^ ^]^

Nanoemulsions are emulsions ≈100 nm, consisting of water, oil, and emulsifier. In addition, nanoemulsions are kinetically stable and can be sterilized under high pressure and temperature. Nanoemulsions are widely applied in cosmetics and drug carriers. Accumulating excessive lipids on DCs could cause aberrantly endoplasmic reticulum (ER) stress and oxidative stress, leading to dysfunctional DCs. Lu et al. generated a KIRA6‐loaded *α*‐tocopherol nanoemulsion that can inhibit X‐box binding protein 1 (XBP1) and ROS‐induced lipid accumulation in DCs. Consequently, the refunctioned DCs can expand and stimulate cytotoxicity T cells, boosting the anti‐tumor immune responses.^[^ ^130^ ^]^ Insufficient activation of the MHC system limits the efficacy of cancer immunotherapy. Shi et al. fabricated mRNA‐ and protein‐based antigenic platforms by combining nanoemulsion with full‐length tumor model antigen OVA. Under the stimulation of mRNA or/and proteins, the DCs were activated with MHCI and MHCII‐based immune responses.^[^ ^131^ ^]^ The hypoxia TME limits current attempts at reprogramming M2 to M1 macrophages. Jiang et al. encapsulated KIRA6 into nanoemulsion to inhibit the ER stress and oxidative stress. As a result, even under the hypoxia TME, the M2 macrophages were reprogrammed by the increasing glycolysis and suppressing fatty acid oxidation (FAO), which could also be a sensitizer for the anti‐PD‐1 treatment.^[^ ^132^ ^]^

Since the first cell membrane‐based nanoparticles were reported by Zhang et al., increasing attention has been attracted by the cell membranes, exosome, bacterial, and virus‐associated nanotechnologies due to the unique innate bioactivity of these lipids.^[^ ^133^ ^]^ By utilizing the tumor‐associated antigens on the cancer cell membranes, Cheng et al. fused 4T1 cancer cell membranes with commercial lipids and monophosphoryl‐lipid A (MPLA) to fabricate a cancer vaccine. After 1 month of intravenous injection with cancer vaccines, the BALB/c mice were challenged with subcutaneous injection with 4T1 cancer cells. The immunized mice exhibited an increased percentage of effector memory T cells that were differentiated from the CD8^+^ T cells after stimulating by antigens for long‐term memory; in addition, the immunized mice had a prolonged survival period.^[^ ^134^ ^]^ Similarly, Li et al. combined photothermal therapy with weak‐immunostimulation in treating breast cancer. 4T1 cancer cell membranes were coated on the surface of porous silicon@Au NPs, followed by immunizing the mice. There were no solid tumors and the survival period of immunized mice was 55 days. In addition, the NPs could significantly inhibit the growth and metastasis of established solid tumors with the effect of photothermal therapy and immunotherapy.^[^ ^135^ ^]^

**Figure 7:**
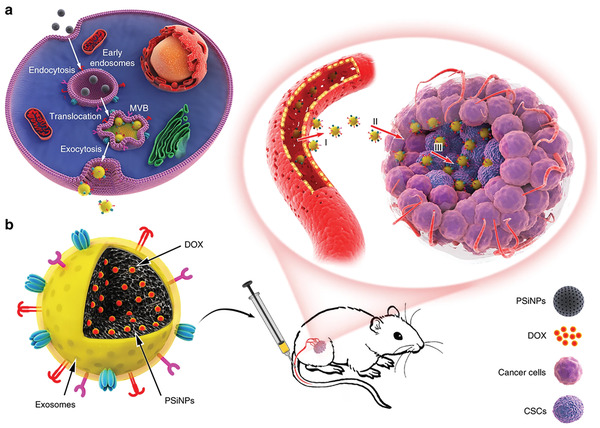
Schematic illustration of E‐PSiNPs as drug carriers for targeted cancer chemotherapy. a) Schematic illustration of the preparation of DOX@E‐PSiNPs. DOX@PSiNPs are endocytosed into cancer cells after incubation, then localized in multivesicular bodies (MVBs) and autophagosomes. After MVBs or amphisomes fuse with cell membrane, DOX@E‐PSiNPs are exocytosed into extracellular space. b) Schematics showing how DOX@E‐PSiNPs efficiently target tumor cells after intravenous injection into tumor‐bearing mice. b‐i) DOX@E‐PSiNPs efficiently accumulate in tumor tissues, b‐ii) DOX@E‐PSiNPs penetrate deeply into tumor parenchyma, and b‐iii) DOX@E‐PSiNPs are efficently internalized into bulk cancer cells and cancer stem cells (CSCs) to produce strong anticancer efficacy. Reproduced with permission.^[^ ^137^ ^]^ Copyright 2019, Springer Nature.

Besides cell membranes, exosomes, as membrane‐bound extracellular vesicles, have specialized functions. Zhang et al. isolated exosomes from neutrophils; and then, modified these exosomes with superparamagnetic iron oxide nanoparticles (SPION‐Ex) to guide these NPs arriving at the tumor. The neutrophil‐derived exosomes can induce cancer cell apoptosis by activating the caspase‐related pathways and delivering the cytotoxic proteins. Compared to the DOX‐loaded liposomes, neutrophils‐derived exosomes, and DOX‐loaded neutrophils‐derived exosomes, the SPION‐Ex increased the NPs accumulation in tumors with improved inhibition of tumor cell proliferation.^[^ ^136^ ^]^ As shown in **Figure** **7**, Yong et al. incubated DOX‐loaded porous silicon NPs (DOX@PSiNPs) with cancer cells to prepare the exocytosed exosomes‐sheathed (E‐PSiNPs). After sheathing with exosomes, NPs exhibited enhanced tumor accumulation, extravasation from blood vessels, and deep penetration into tumor parenchyma. Moreover, a prolonged survival period was observed in the orthotopic 4T1 tumor‐bearing mice, H22 tumor‐bearing mice, and B16‐F10 lung metastasis mice treated with NPs.^[^ ^137^ ^]^

By taking advantage of bacterial immunogenicity, Patel et al. extracted bacterial membranes from Mycobacterium smegmatis; then, CpG (TLR9 agonist) and pH‐responsive polymer PC7A (endosome disruption) were coated with the bacterial membranes for a further immune boost. Together with radiotherapy, the bacterial membrane could capture the cancer neoantigens and enhance the uptake in DCs, followed by improved cross‐presentation to T cells. Tumor‐specific antitumor immune memory could be observed in melanoma and neuroblastoma‐bearing mice treated with the NPs.^[^ ^138^ ^]^

The virus is non‐self, which could trigger the immune response due to its inherent adjuvant properties. Especially, oncolytic viruses can only replicate in cancer cells, leading to the lysis of cancer cells rather than that of the normal cells. Fusciello et al. coated oncolytic adenovirus serotype 5 with A549 cell membranes to prepare the artificially cloaked viral nanovaccines. In both B16F10 and A549‐bearing mice, the nanovaccines induced DCs cross‐presenting the tumor‐specific antigen SIINFEKL on their surface, leading to the increased priming of the effector T cells. In addition, the nanovaccine could also function as a preventive vaccination in protecting against tumor challenges, controlling tumor growth, and prolonging the survival period.^[^ ^139^ ^]^

### 4.3 Inorganic‐Based NPs

Compared to polymer‐based NPs and lipid‐based NPs, inorganic‐based NPs are highly stable. Inorganic‐based NPs are commonly divided into gold NPs, magnetic NPs, black phosphorus NPs, and silicon NPs. These inorganic‐based NPs also exhibit unique, innate properties, such as photothermal and imaging capabilities, which are widely applied in tumor diagnosis, imaging, and therapy.^[^ ^140^ ^]^

Gold NPs are usually synthesized by reducing the hydrogen tetrachloroaurate (HAuCl_4_) with the stabilizing agent. Gold NPs are characteristic of electrical conductivity, surface plasmon resonance, redox activity, and fluorescence quenching. Su et al. modified polyaniline‐based glyco structures on the surface of Au NPs to polarize M2 to M1 macrophages. The Au NPs with smaller sizes exhibited a higher uptake efficiency on M2 macrophages than the larger ones. In addition, after uptake by macrophages, Au NPs also enhanced ER stress induction and activation of the spleen tyrosine kinase signaling pathway, leading to the reprogramming of TAMs in lung cancer models with no apparent toxicity.^[^ ^141^ ^]^ Gao et al. coated *β*‐cyclodextrin (*β*‐CD)–modified Au NPs and adamantane (ADA)–modified Au NPs with the outer membranes of Escherichia coli. Bacterial membranes triggered the phagocytosis of Au NPs by circulating immune cells, leading to the intracellular degradation of Au NPs and supramolecular self‐assembly of Au NPs driven by *β*‐CD–ADA host–guest interactions. This process turned dispersed Au NPs without photothermal effect into Au NPs aggregates with photothermal effect. Moreover, the immune cells transported these Au NPs to the tumor; together with the photothermal treatment, Au NPs induced the tumor cells' apoptosis, releasing the inflammatory tumor signals and recruiting more immune cells to the tumor.^[^ ^142^ ^]^

Magnetic NPs can be synthesized from either top–down or bottom–up methods; in addition, the size of magnetic NPs can be controlled between 10 and 00 nm. A key advantage of magnetic NPs is that the movement of these NPs can be precisely controlled under an external magnetic field. In addition, magnetic NPs can distort the magnetic field in their surroundings, forming the basis for enhanced contrast in magnetic resonance imaging (MRI). Magnetic NPs can also be photothermal by converting lighting energy into thermal energy. Li et al. integrated ultrasmall iron oxide nanoparticles (UIONPs) with poly (ethylene glycol)‐block‐poly(2‐hexamethylenediamine) ethyl methacrylate (PHMA) and red blood cell membranes into one single platform. The NPs could be delivered into the tumor site by passive targeting delivery, activating the stimulator of the interferon genes (STING) pathway with the secretion of type‐I interferon (IFN‐*β*) in TAMs. The IFN‐*β* attracted the surrounding DCs, and the DCs further primed the tumor‐specific CTLs. Combing with the immune checkpoint block therapy, the NPs remarkably inhibited tumor growth and prolonged melanoma and breast tumor‐bearing mouse models' survival.^[^ ^143^ ^]^ Zhao et al. carefully prepared yolk‐shell NPs consisting of Fe_3_O_4_ core with MnO_2_ shell modified with carbon shell and cationic polymer CD‐PGEA, loading with p53. The multifunctional NPs could enhance the T1–T2 dual‐mode MRI and induce the ICD by depleting the GSH, photothermal effect, and p53 plasmid delivery. After intravenous injection, the NPs accumulated in the tumor, as proven by the T1–T2 dual‐mode MRI; the NPs also exhibited inhibited effects in both local and metastatic 4T1 tumor‐bearing mice.^[^ ^144^ ^]^ Howard et al. isolated nanomagnets from specialized magnetotactic bacteria, followed by combing them with oncolytic viruses (HSV1716) to prepare the NPs. Under the magnetic field, nanomagnets could shield the HSV1716 from neutralizing antibodies and increase the local concentration of HSV1716 in the tumor by magnetic targeting. Furthermore, the NPs could boost the T cell‐mediated immune responses converting the cold tumor into a hot one.^[^ ^145^ ^]^

2D materials exhibit broad, unique properties, such as conductivity, thermal conductivity, optoelectronics, energy savings, and catalysis. Among these 2D materials, ultrathin black phosphorus monolayers attract wide attention owing to the unique layered structure and layer‐dependent band gap. Particularly, phosphate is an essential element that counts for 1% of total body mass; phosphorous is also a critical component of nucleic acids. Therefore, black phosphorus is biocompatible with potential applications in the biomedical field.^[^ ^146^ ^]^ Li et al. modified ultra‐small black phosphorus with PEG, polyacrylic acid (PAA) to capture Ag^+^ ions and polypropylene sulfide (PPS). With the coordination of Ag^+^, the modified black phosphorus could be self‐assembled into NPs with the PPS as the shell structure and the other as the core. The NPs enhanced the light absorption in the NIR‐II region by integrating black phosphorus and Ag^+^. In addition, the Ag^+^‐mediated pro‐inflammatory immune responses during the PDT process induced the ICD in tumor immunological models, causing the activation of CTLs. Consequently, tumor growth and metastasis were inhibited by the therapeutic effects of NPs.^[^ ^147^ ^]^ Zhang et al. coated the black phosphorus with PEGylated hyaluronic acid as multifunctional NPs. Then, these NPs were delivered to the tumor site by CD44‐mediated active targeting and EPR‐mediated passive targeting strategies. Under the combination of PTT and PDT, the expression of CD206 in macrophages was decreased by 42.3%, with a 59.6% increased expression of CD86. Moreover, ICD and released damage‐associated molecular patterns (DAMPs) could be observed in the tumor site with the maturation of DCs and boosting of CTLs.^[^ ^148^ ^]^ Ou et al. functionalized active photosynthetic Chlorophyceae with black phosphorus by interacting with polyaspartic acid (PASP) and Fe^3+^. Under the stimulation of light, the NPs generated oxygen in the tumor site, ameliorating the tumor hypoxia, consuming the GSH, and inducing the Fenton reactions and ICD. Furthermore, the NPs increased the infiltration of the immune cells and stimulated the proliferation and maturation of immune cells.^[^ ^149^ ^]^

Porous silicon NPs are usually prepared by the top–down method. Electrochemical etching is applied to process the single crystalline silicon wafer to the porous silicon NPs with a diameter from 60 to 500 nm.^[^ ^150^ ^]^ In addition, the surficial functional groups of porous silicon NPs are silicon hydride (Si—H), silanol (Si—OH), and siloxane (Si—O—Si). Furthermore, porous silicon NPs can be degraded into silicic acid, indicating the biocompatibility and biodegradability of porous silicon NPs.^[^ ^151^ ^]^ Kim et al. prepared porous silicon NPs with high loading efficacy of SiRNA coated with fusogenic membranes. The NPs could first sensitize cancer cells to cisplatin and then reprogram the TAMs to inhibit their oncogenic pathways by down‐regulating the expression of PI3k*γ*, which further recruited the CTLs to the tumor and sensitized immunosuppressive myeloid cells to checkpoint inhibitors.^[^ ^152^ ^]^ Stead et al. modified porous silicon with anti‐CD209 antibody loading with sirolimus. Due to the modification of anti‐CD209, the NPs platform was phagocytosed by monocyte‐derived and myeloid DC in whole human blood in a time‐ and dose‐dependent manner. In addition, NPs contributed to a maturation‐resistant phenotype and significantly suppressed allogeneic T‐cell proliferation.^[^ ^153^ ^]^ Shahbazi et al. cultured various cells, such as B lymphocytes (Raji), T‐cells (Jurkat), monocytes (U937), and RAW 264.7 macrophage cells, with five different porous silicons, such as thermally oxidized PSi (TOPSi), thermally carbonized PSi (TCPSi), (3‐Aminopropyl) triethoxysilane functionalized thermally carbonized PSi (APSTCPSi), thermally hydrocarbonized PSi (THCPSi), and undecylenic acid functionalized THPSi (UnTHCPSi), at a similar size, surface area, and pore volume. They systemically explored the immunotoxicity of these porous silicon NPs; THCPSi and UnTHCPSi NPs were more immunogenic than other porous silicon NPs.^[^ ^154^ ^]^

### 4.4 Summary

With the development of NPs and nanomedicine, researchers have provided promising strategies for cancer immunotherapy. The commonly used NPs systems are polymer‐based NPs, lipid‐based NPs, and inorganic NPs. The tunable physicochemical properties, such as hydrodynamic diameter, surficial charge, morphology, and hydrophilicity, can be utilized to prepare the NPs with different affinities to the tumor cells. In addition, the ERP effects and active targeting capabilities contribute to the increasing NPs concentration in tumor tissues due to the abnormal vascular structures. Moreover, NPs are generally applied as drug carriers under various stimulations, such as GSH, acid pH, NIR light, and magnetic field; the payload can be precisely released on tumor cells or even specific organelles. The released payload can further regulate the TME and improve the potential efficacy of cancer immunotherapy. Together with the NPs, the efficacy of some traditional treatments, such as chemotherapy, radiotherapy, photodynamic and photothermal therapies, and immunotherapy, can be improved.

However, there are still some concerns about applying NPs for cancer immunotherapy. For example, after intravenous injection, oral administration, or inhalation, the biodistribution of NPs should be carefully investigated in the body system, especially in the tumor. This is because most NPs tend to accumulate in the liver, spleen, and kidney, which could decrease the potential therapeutic effects of NPs. In addition, after accumulation in some main organs, such as the lung, heart, liver, or even brain, the potential side effects of NPs should be systematically explored. Moreover, the NPs can be metabolized with time; so, the optimized balance between NPs degradation and therapeutic duration should be thoughtfully decided.

## 5 Conclusion and Future Perspectives

Chemoresistance, serious side effects, insufficient immune cells infiltration, decreased leukocyte caused by the radiotherapy, and limited penetration of photothermal or photodynamic therapy, limit the tumor therapeutic effects. Various factors contribute to unsatisfactory cancer treatments, and one of the essential elements is TME. The dynamic and highly heterogeneous TME consists of not only tumor cells but also of MDSCs, TAMs, DCs, TAFs, ECs, secreted cytokines, and extracellular matrix. The dynamic interactions among these TME compositions make TME a promising target for cancer immunotherapy.

As a result of the unique advantages such as enhanced permeability and retention effects on the tumor, high drug loading efficacy, and stimulation‐responsive properties, NPs are widely applied as therapeutic agent carriers, diagnostic probes, contrast reagents, vaccines, and immune modulators in regulating the TME and cancer immunotherapy. Many studies confirm that NPs can amplify the effects of immune checkpoint blockade therapy, such as CTLA‐4 and PD‐1. Moreover, with the modification of stimulation‐responsive materials and targeting ligands, the NPs tend to accumulate in tumors and release the payload under the various stimulations, such as acid pH, high GSH, and H_2_O_2_, which could cause cascading consequences, such as remodeling TME, inhibited tumor growth, and metastasis. Furthermore, some targeting NPs such as SGT‐53, CALAA‐01, MM‐302, and MCC‐465 are under the phase trials. For example, the wild‐type p53 gene (plasmid DNA)‐loaded cationic liposomes with an anti‐transferrin receptor single‐chain antibody fragment (SGT‐53) were combined with various chemotherapeutic drugs, such as temozolomide, doxorubicin, and gemcitabine, for treating different cancers. As for the MCC‐465, it lost the funding for the following phase trials. Although the targeting and payload were effective, CALAA‐01 exhibited toxicities in the dose‐escalation studies. Moreover, patients treated with MM‐302 exhibited no benefit over the comparator, indicating that the efficiency of MM‐302 was limited. Therefore, there is still a considerable gap between the lab work and the clinical side because the animal experiment cannot detailedly mimic the whole situation in humans; in addition, the researcher still needs to be clear about how the animal immune system works against the tumor. Meanwhile, the researcher is still determining whether the animal immune system works in the same way as humans.

In addition, after the intravenous injection, the surface of NPs could be covered by the protein corona in the bloodstream, quenching the targeted effects. Moreover, for the stimulation‐responsive NPs, most of the stimulation‐responsive drug releases are explored in vitro; it is difficult to confirm that the drug release behavior in vivo would be the same as that in vitro. In addition, it also is challenging to prove that the loaded drug is not released before the NPs arrive at the desired area. Furthermore, the biodistribution, degradation, and biocompatibility of NPs are always the concerns when these NPs are applied in vivo. There are still many challenges to be carefully considered before the NPs can go through the clinical trial.

In summary, smart nanoparticle‐based platforms have shown great potential in regulating TME and enhancing cancer treatments due to their stimulation‐responsive properties and targeting abilities. The inhibited tumor growth or prolonged survival period is associated with responsive drug release and NPs’ accumulation in the tumor site, followed by the regulated TME and immune responses. Applying smart nanoparticle‐based platforms in cancer immunotherapy is still in its infancy, partially due to the abovementioned challenges. Therefore, the researcher is supposed to develop suitable tumor models to mimic real‐world cases in humans in vivo instead of using experimental animals. In addition, the size, surficial charge, hydrophobicity, shape, and PEG chains of NPs would influence the accumulation of NPs in the tumor site. Thus, NPs with precisely controlled parameters can increase the concentration of NPs in the tumor site via the EPR effects.

## Conflict of Interest

The authors declare no conflict of interest.
